# Acquisition of aneuploidy drives mutant p53-associated gain-of-function phenotypes

**DOI:** 10.1038/s41467-021-25359-z

**Published:** 2021-08-31

**Authors:** Lindsay N. Redman-Rivera, Timothy M. Shaver, Hailing Jin, Clayton B. Marshall, Johanna M. Schafer, Quanhu Sheng, Rachel A. Hongo, Kathryn E. Beckermann, Ferrin C. Wheeler, Brian D. Lehmann, Jennifer A. Pietenpol

**Affiliations:** 1 Department of Biochemistry, Vanderbilt University, Nashville, TN USA; 2 Inscripta, Inc, Boulder, CO USA; 3 Vanderbilt-Ingram Cancer Center, Vanderbilt University Medical Center, Nashville, TN USA; 4 Pelotonia Institute for Immuno-Oncology, The James Comprehensive Cancer Center, The Ohio State University, Columbus, OH USA; 5 Department of Biostatistics, Vanderbilt University Medical Center, Nashville, TN USA; 6 Department of Medicine, Vanderbilt University Medical Center, Nashville, TN USA; 7 Department of Pathology, Microbiology and Immunology, Vanderbilt University Medical Center, Nashville, TN USA

**Keywords:** Tumour-suppressor proteins, Oncogenes

## Abstract

p53 is mutated in over half of human cancers. In addition to losing wild-type (WT) tumor-suppressive function, mutant p53 proteins are proposed to acquire gain-of-function (GOF) activity, leading to novel oncogenic phenotypes. To study mutant p53 GOF mechanisms and phenotypes, we genetically engineered non-transformed and tumor-derived WT p53 cell line models to express endogenous missense mutant p53 (R175H and R273H) or to be deficient for p53 protein (null). Characterization of the models, which initially differed only by *TP53* genotype, revealed that aneuploidy frequently occurred in mutant p53-expressing cells. GOF phenotypes occurred clonally in vitro and in vivo, were independent of p53 alteration and correlated with increased aneuploidy. Further, analysis of outcome data revealed that individuals with aneuploid-high tumors displayed unfavorable prognoses, regardless of the *TP53* genotype. Our results indicate that genetic variation resulting from aneuploidy accounts for the diversity of previously reported mutant p53 GOF phenotypes.

## Abstract (web summary)

Previous studies report that mutant p53 proteins have gain-of-function activities and cause oncogenic phenotypes. Herein, the authors engineered two isogenic epithelial cell lines to express wild-type or missense mutant p53 or be deficient for p53 protein and show that aneuploidy drives several of the GOF phenotypes previously ascribed to mutant p53.

## Introduction

Mutation of *TP53* is one of the most frequent genomic alterations in human tumors. *TP53* encodes p53, a sequence-specific transcription factor that regulates gene expression involved in numerous cellular processes, including maintenance of genome stability^1^. Mutations in p53 are frequently single amino acid missense mutations that alter the structure of the DNA-binding domain or affect residues that directly contact DNA, both of which lead to a loss of function (LOF) in p53 DNA binding and regulation of gene expression^2,3^. In addition, several high-frequency “hotspot” missense mutations have been proposed to confer oncogenic gain-of-function (GOF) properties^4,5^. Many p53 GOF phenotypes have been described using in vitro cell model systems and mouse models, including increased proliferation^6^, colony formation^7,8^, genomic instability^9–11^, metastasis^6^, xenograft growth^4,8^, metabolic dysregulation^12–14^, enhanced migration^15–18^, and development of distinct tumor spectra^19^.

Understanding mutant p53 GOF activities is complicated by the diversity and context-specific nature of reported GOF phenotypes. The study of mutant p53 GOF activities is made even more challenging because mutations in p53 are positively correlated with the development of aneuploidy^20–22^, which can increase heterogeneity through diverse chromosomal alterations and contribute to tumorigenesis^23–26^. Recent advances in genome editing enable the generation of model systems that can circumvent confounding experimental limitations of overexpression models, including nonphysiological protein expression lacking regulation by the endogenous promoter. Use of the CRISPR/Cas9 system allows for the study of mutant p53 function in a controlled context and has led to observations that challenged the mutant p53 GOF hypothesis in myeloid malignancies^27^. Given the potential of using mutant p53 protein as a therapeutic target in many cancer types, and the longstanding debate of whether GOF activities exist, there is a critical need to study mutant p53 GOF activities in models of epithelial malignancies controlled for the confounding effects of exogenous protein expression and chromosomal alterations that occur following p53 LOF.

In this study we used CRISPR/Cas9-mediated genome editing and developed two isogenic epithelial cell line models (one nontransformed and one tumor-derived). Parental cells were modified to express either of the most frequently occurring p53 missense mutations (R175H or R273H), to be deficient for p53 protein (null), or to retain the wild-type (WT) protein. Endogenous p53 expression is regulated by the native p53 promoter in these engineered models, providing a biologically relevant system for rigorous functional experimentation across different p53 states. Additionally, the use of clonally derived cell lines originating from the same near-diploid parental genetic background allows for assessment of the genomic alterations and resulting molecular heterogeneity following mutation of *TP53*. Through our comparative analyses of isogenic epithelial cells, which initially differed only by *TP53* genotype, we discovered that increased aneuploidy is consistently observed in our mutant p53-expressing cell lines. Further, we discovered that in vitro GOF phenotypes are only present in mutant p53-expressing cell lines that display increased aneuploidy and that these phenotypes are not dependent on mutant p53 protein expression. Mutant p53-containing cell lines did not have increased tumorigenicity or metastasis in vivo, and analysis of human tumors revealed that loss of p53 function and increased aneuploidy were associated with unfavorable prognoses. Importantly, our results reveal aneuploidy as a mechanism contributing to the diversity of reported mutant p53 GOF phenotypes.

## Results

### Generation and characterization of genetically engineered cell line models to study potential mutant p53 GOF activities

**Fig. 1:**
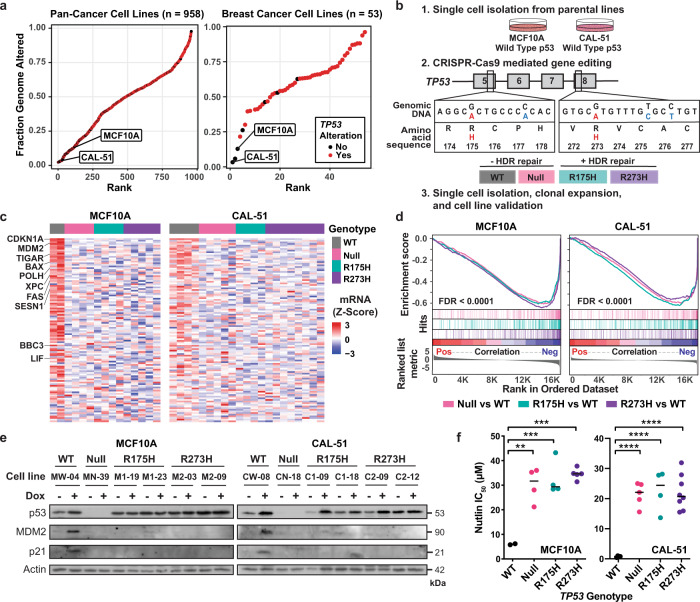
Generation and characterization of genetically engineered epithelial cell line models to study potential mutant p53 GOF activities. **a** Fraction of the genome altered across pan-cancer (*n* = 958, left panel) and breast cancer cell lines (*n* = 53, right panel) from the Cancer Cell Line Encyclopedia, with (red) and without (black) *TP53* alterations (mutation or deletion); including the nontransformed MCF10A cell line. **b** Experimental workflow for CRISPR-Cas9 genetic engineering of isogenic cell line models with *TP53* missense (red) and synonymous (blue) mutations and the resulting isogenic cell lines that either did (R175H and R273H, +HDR) or did not undergo complete homology-directed repair (WT and Null, −HDR). HDR homology-directed repair. **c** Heatmap of normalized gene expression for the top 116 p53 target genes^33^ for all cell lines at passage five after clonal expansion. **d** GSEA plot showing negative enrichment of p53 target genes (Fischer Direct p53 Targets Meta Analysis geneset) from RNA-seq differential gene expression analyses between *TP53* Null (pink), R175H (teal), and R273H (purple) clones compared to the MCF10A and CAL-51 WT cell lines. Pos positive, Neg negative, FDR false discovery rate. **e** Western blots of relative p53, MDM2, p21, and actin protein levels in the indicated cell lines after 6 h of doxorubicin treatment (dox, 0.2 µM). Western blots of additional cell lines are shown in Supplementary Fig. 1b, c. Blots are representative of two independent experiments. **f** IC_50_ values for Nutlin-3a in the MCF10A (*n* = 2 WT, 4 Null, 4 R175H, and 5 R273H) and CAL-51 (*n* = 4 WT, 5 Null, 4 R175H, and 8 R273H) cell lines after treatment for 72 h. Dots represent the mean IC_50_ per cell line calculated from at least two independent experiments and black lines indicate median IC_50_ per *TP53* genotype. One-way ANOVA with Dunnett’s multiple comparison test, **P < 0.01, ***P < 0.001, ****P < 0.0001. Source data and exact *P* values are provided in the Source Data File.

To select cell lines that would provide controlled model systems for the study of p53 mutation, we evaluated p53 status and the fraction of the genome altered (FGA) across cancer cell lines (Cancer Cell Line Encyclopedia, *n* = 957). We selected two *TP53* WT lines derived from breast epithelium that are near-diploid and have a low FGA (MCF10A = 13%, CAL-51 = 3%) compared to pan- or breast cancer cell lines (Fig. 1a and Supplementary Fig. 2a). MCF10A is a nontransformed cell line derived from normal human mammary epithelium^28^. CAL-51 is a metastatic breast cancer cell line derived from a pleural effusion^29^. Both lines are considered “triple-negative” since they lack estrogen and progesterone receptor expression and *HER2* amplification^30,31^. To minimize genomic heterogeneity, we performed single-cell selection and clonal expansion to generate a parental clonal line for each model. Using CRISPR/Cas9-mediated gene editing^32^, we generated mutations in *TP53* using homology-directed repair (HDR) templates containing mutant bases corresponding to hotspot *TP53* mutations, R175H and R273H, along with synonymous mutations to prevent CRISPR/Cas9-mediated cleavage of the HDR-recombined alleles (Fig. 1b, and Methods). As controls for CRISPR-mediated off-target effects, we identified WT cell lines that underwent incomplete HDR (containing engineered synonymous mutations but not the missense mutation). *TP53* null cell lines were generated from frameshift insertions or deletions in both alleles, resulting in cells deficient for full-length p53 protein (Supplementary Table 1). Through single-cell isolation, clonal expansion, and genotyping, we derived independent biological replicates for each model and *TP53* genotype; WT (R/R), null (−/−), and the two p53 mutants, R175H and R273H (H/−, or H/H) (Fig. 1b, Supplementary Fig. 1a, Supplementary Table 1). In total, we generated 15 clonally derived isogenic MCF10A cell lines (2 WT, 4 null, 4 R175H, and 5 R273H) and 21 clonally derived isogenic CAL-51 cell lines (4 WT, 5 null, 4 R175H, and 8 R273H) (Supplementary Fig. 1a).

To analyze p53 activity in the engineered *TP53* lines, we performed RNA sequencing (RNA-seq) on all cell lines derived from MCF10A and CAL-51 models. Both models displayed decreased p53 target gene expression in the *TP53* null, R175H, and R273H mutant cell lines compared to WT cell lines^33^ (Fig. 1c). Geneset enrichment analysis (GSEA) showed significant negative enrichment of p53 target gene expression in R175H and R273H mutant and null cell lines (all comparisons: NES < −2.8, FDR < 0.0001) (Fig. 1d), demonstrating p53 LOF as a sequence-specific transcription factor in the null, R175H and R273H cell lines. We also analyzed protein expression after exposure of the MCF10A and CAL-51 models to the anthracycline doxorubicin. Doxorubicin treatment resulted in increased p53 protein levels and downstream targets, MDM2 and p21, in cell lines expressing WT p53. p53 protein levels also increased after doxorubicin treatment in mutant cell lines in the transformed CAL-51 model, similar to previous reports^27,34^. However, no changes in MDM2 or p21 expression were observed, further indicating p53 LOF in null, R175H, and R273H p53 mutant cell lines (Fig. 1e, Supplementary Fig. 1b, c). To further analyze p53 activity, we treated all cell lines with the small molecule Nutlin-3a, which inhibits the interaction between p53 and MDM2, the primary p53 negative regulator^35^. Comparison of half-maximal inhibitory concentrations (IC_50_) revealed that only WT cell lines were sensitive to Nutlin-3a, while all null, R175H, and R273H cell lines showed a significant increase in IC_50_ (null, R175H, and R273H compared to WT: MCF10A, *P* < 0.0015; CAL-51, *P* < 0.0001) (Fig. 1f). Therefore, genome editing of WT *TP53* to null, R175H, or R273H mutations abrogated canonical p53 function and resulted in a loss of sensitivity to Nutlin-3a, consistent with previously reported results^27,36^.

### p53 mutant isogenic cell lines display increased frequency of aneuploidy

**Fig. 2:**
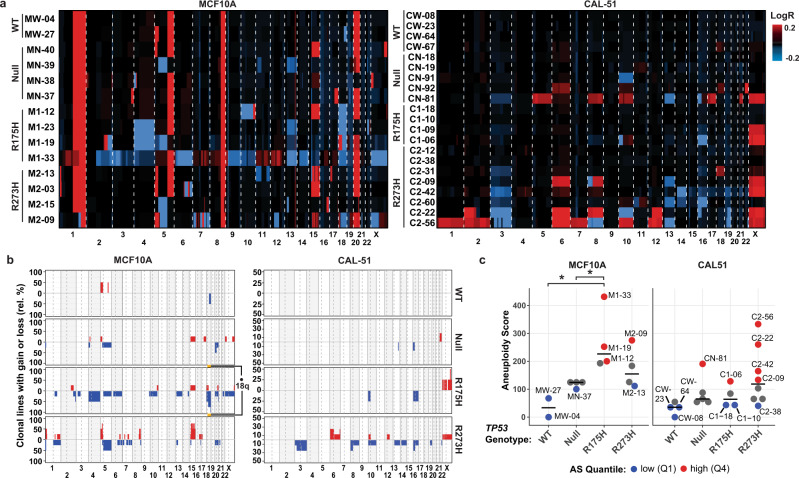
p53 mutant isogenic cell lines display increased frequency of aneuploidy. **a** Copy number alterations (Log_2_ ratios, LogR) in MCF10A (left) and CAL-51 (right) isogenic cell lines grouped by *TP53* genotype. Chromosomal gain (red) and loss (blue). **b** Frequency plots of the proportion of clonal lines from each genotype containing the indicated chromosomal copy number gains (red) or losses (blue). Chromosomal alterations were calculated relative to the parental WT clone in the MCF10A (left) and CAL-51 (right) cell lines from **a**. Chromosome 18q in the R175H mutant MCF10A cells was the only region significantly altered; • adjusted *P* < 0.1, two-sided Student’s *t*-test comparing chromosome arms between *TP53* genotypes. **c** Aneuploidy score (AS) in each MCF10A (left) and CAL-51 (right) cell line by *TP53* genotype (MCF10A *n* = 2 WT, 4 Null, 4 R175H, and 4 R273H cell lines; CAL-51 *n* = 4 WT, 5 Null, 4 R175H, and 8 R273H cell lines). Colors indicate cell lines classified as aneuploid-low (lower quantile AS [Q1], blue), and aneuploid-high (upper quantile AS [Q4], red). Cell lines colored in gray indicate an intermediate AS (quantiles 2 and 3). *P < 0.05 (WT vs R175H, *P* = 0.011; Null vs R175H, *P* = 0.046), one-way ANOVA with Dunnett’s multiple comparison test. Source data are provided in the Source Data File.

Mutation of p53 is associated with development of aneuploidy^9,37^. To evaluate the relationship between p53 mutations and the development of aneuploidy, we assessed DNA content in our MCF10A and CAL-51 models early (passage five) after clonal expansion. Analysis of metaphase spreads and flow cytometry of propidium iodide (PI)-stained cells revealed that while the MCF10A derived lines maintained a near-diploid median number of chromosomes, five of the CAL-51 lines (three R273H, one R175H, and one null) contained a median number of chromosomes >2 N (Supplementary Fig. 2b, c). Four of these five were nearly tetraploid, suggesting that whole-genome doubling (WGD) likely occurred in these cells. Since metaphase spreads and PI staining detect gross chromosomal alterations, we also examined DNA copy number by cytogenomic microarray analysis (CMA) after 30 passages in culture. All MCF10A cell lines showed aneuploidy, with chromosomal gains (1q, a portion of 5q and 8q, and 20) consistent with known parental MCF10A cell line karyotypes (Fig. 2a and Supplementary Fig. 2a). Additional whole-chromosome or arm-level alterations were identified in chromosomes 4, 5, 13, 15, and 18 (Fig. 2a). Structural alterations, including gain or loss of chromosomal regions, were evident in all MCF10A cell lines. Chromothripsis, a chromosome shattering and rearrangement event that manifests as alternating patterns of gains and losses, was also detected in several MCF10A R273H mutant clones (M2-09, M2-03, and M2-13, Fig. 2a; chromosomes 6, 7, and 15, Supplementary Fig. 3). In contrast, the CAL-51 cells displayed mostly whole-chromosome alterations, such as gains in chromosomes 6 and X and losses in chromosomes 3, 13, and 16 in both the *TP53* mutant and null genotypes (Fig. 2a), consistent with other analyses of tetraploid cells^38^.

To identify chromosomal alterations common to each genotype, we generated frequency plots to show the percentage of cell lines with a given chromosomal alteration for each *TP53* genotype relative to the parental WT cell line. Compared to null cells, mutant cell lines in both models appeared to have increased frequency of chromosomal gains and losses (Fig. 2b). Statistical analysis to compare differentiated chromosomal arm regions between mutant and null cells revealed that chromosome 18q in the R175H mutant MCF10A cells was the only region significantly altered (adjusted *P* < 0.1). To quantify the degree of copy number alterations within each cell line, we generated an integrative aneuploidy score (AS) from the CMA data that reflects the sum of segmented copy number alterations (Log_2_ ratios) in the clonal lines relative to the respective parental WT clone. In both models, mutant and null cell lines showed an increased median AS compared to WT (MCF10A R175H *P* = 0.011). Compared to null lines, there was a significant increase in AS in MCF10A R175H lines (*P* = 0.046) (Fig. 2c). The median AS was higher in the R273H mutant compared to null lines in both models, although not reaching statistical significance (Fig. 2c). For use in subsequent comparative analyses of the models, AS quantiles were used to classify cell lines in each model as aneuploid-high (upper quantile AS) or aneuploid-low (lower quantile AS) (Fig. 2c).

To examine the possibility that acquired mutations in other genes could account for the observed increased aneuploidy in our models, we conducted whole-exome sequencing (WES) in tandem with CMA. No gene mutations were significantly enriched in aneuploid-high cell lines in either model (Supplementary Table 2). Further, no significant enrichment in mutations was observed for a set of genes known to be associated with hereditary cancers (Supplementary Fig. 4a)^39^. However, the MCF10A cell line with the highest AS (M1-33) displayed deletions in *BRCA2* and *RB1* (Supplementary Fig. 4a). There was not a significant difference in the total number of mutations in either model relative to *TP53* genotype (Supplementary Fig. 4b). In sum, these results show that aneuploidy occurs at an increased frequency after *TP53* mutation in both isogenic models.

### Gene expression changes are associated with aneuploidy and not mutant p53 expression

**Fig. 3:**
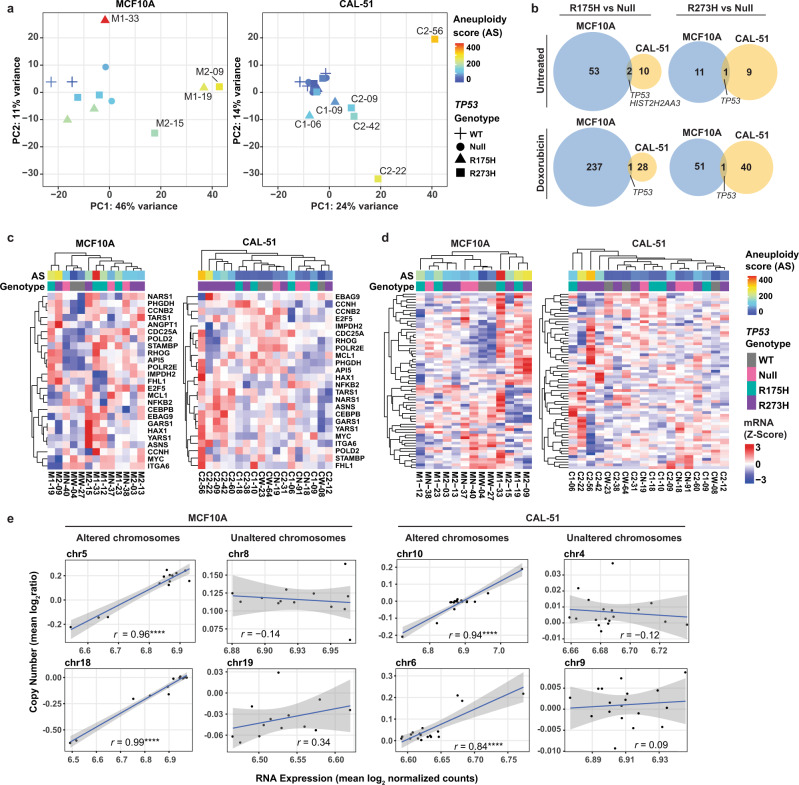
Gene expression changes are associated with aneuploidy and not mutant p53 expression. **a** Principal component (PC) analysis of gene expression values for untreated MCF10A (left panel) and CAL-51 (right panel) isogenic cell lines. **b** Venn diagrams showing the overlap between differentially expressed genes in R175H (left) and R273H (right) cell lines compared to null cells in MCF10A (blue) and CAL-51 (yellow) models. Cells were either untreated (top) or treated with doxorubicin (0.2 µM, 6 h) (bottom). Cell lines used in RNA-seq analyses are shown in Supplementary Fig. 5a. **c** Hierarchical clustering and comparison of gene expression for previously reported mutant p53-associated upregulated genes^41^ from the indicated MCF10A (left panel) and CAL-51 (right panel) cell lines. **d** Hierarchical clustering and comparison of gene expression for the karyotype heterogeneity associated HET70 gene signature^43^ from the indicated MCF10A (left panel) and CAL-51 (right panel) cell lines. **e** Scatter plots comparing average chromosomal copy number with chromosomal RNA expression for MCF10A (left panel, *n* = 13) and CAL-51 (right panel, *n* = 18) cell lines across frequently altered or unaltered chromosomes. Each point represents the mean per cell line. The blue line represents a linear model of the best fit, with the gray area representing the 95% confidence intervals. *r* = Pearson correlation coefficient, ****P < 0.0001, Pearson correlation. Source data are provided in the Source Data File.

Previously, mutant p53 GOF phenotypes were ascribed to the acquisition of a mutant p53-dependent transcriptional program^40–42^. To investigate this possibility in our models, we performed RNA-seq in parallel with CMA and WES experiments on MCF10A and CAL-51 cell lines cultured under normal conditions or with doxorubicin to induce genotoxic stress (Supplementary Fig. 5a). Principal component analyses (PCA) of all genes revealed differences in global gene expression patterns, with cell lines clustering independent of *TP53* genotype or doxorubicin treatment in both models (Fig. 3a and Supplementary Fig. 5b). Further, we found that the most significant degree of variance in the PCA could be attributed to cell lines with higher levels of aneuploidy (Compare Fig. 3a to Fig. 2c). We conducted differential gene expression analyses comparing R175H or R273H mutants to null cells in MCF10A and CAL-51 models. Comparison of differentially expressed genes (DEGs) revealed almost no shared genes between R175H or R273H mutants in both MCF10A and CAL-51 models, except for *TP53* and a gene encoding for Histone H2A (Fig. 3b, Supplementary Data 1). R175H mutant cells in the MCF10A cell line had a larger number of DEGs; however, GSEA revealed that the most significantly altered pathway between MCF10A R175H and null cells was the chromosome positional pathway CHR18Q21 (Supplementary Fig. 5c), indicating that increased DEGs are related to chromosomal changes in those cells (Fig. 2b).

To further assess the impact of aneuploidy on the number of DEGs, we conducted differential gene expression analysis between CAL-51 R273H mutants with varying levels of aneuploidy (high, medium, or low AS) and two aneuploid-low null cell lines (Supplementary Fig. 5d). Mutant clones with the greatest aneuploidy showed the greatest number of DEGs, whereas comparison of R273H AS-low versus null AS-low cells revealed only 17 significantly DEGs (Supplementary Fig. 5e). To test if mutant p53 expression affected the number of DEGs, we generated stable p53 knockdown cell lines from our isogenic models with shRNAs targeting *TP53* or a nontargeting control (NT) (Supplementary Fig. 6a). We validated knockdown of p53 by RNA-seq or western blot (Supplementary Fig. 6b, c). RNA-seq comparing aneuploid-high CAL-51 R273H cell lines (C2-22 and C2-56) with NT control to those with p53 knockdown revealed only six DEGs (Supplementary Fig. 6d), and there were no differences in global gene expression patterns by PCA between the p53 knockdown and NT control cells (Supplementary Fig. 6e). These data suggest that DEGs in our isogenic p53 mutant cell lines result from genetic variation and not a novel transcriptional program induced by mutant p53.

Distinct transcriptional programs have been reported to be transactivated by mutant p53^41^. However, we did not observe genotype-specific patterns for these reported genes, with heterogeneous expression observed across cell lines in both models (Fig. 3c). In the CAL-51 model, unsupervised hierarchical clustering revealed that cell lines with a higher AS clustered together and had increased expression of most of these reported mutant p53-upregulated genes (Fig. 3c, right). To determine if our cell lines displayed gene expression patterns consistent with those previously found in aneuploid cells, we analyzed expression of the HET70 geneset, which consists of genes upregulated in cells displaying karyotype heterogeneity^43^. Unsupervised hierarchical clustering revealed that cell lines with a higher AS clustered together in both MCF10A and CAL-51 models and had increased expression of HET70 genes (Fig. 3d). These data suggest transcriptional changes are associated with karyotype heterogeneity and are not driven by the p53 mutations analyzed herein.

We hypothesized that aneuploidy and the resulting stochastic genetic variation led to the altered gene expression profiles in *TP53-*mutated cells. To test this hypothesis, we determined if gene dosage effects resulting from chromosomal alterations aligned with the changes in gene expression observed in the PCA (Fig. 3a). For each untreated cell line, we compared the average transcriptome expression per chromosome to the average chromosomal copy number and observed a significant correlation in highly altered chromosomes across cells from both models (MCF10A, chromosome (chr) 5: r = 0.96, chr18: r = 0.99; CAL-51, chr10: r = 0.94, chr6: r = 0.84; all *P* < 0.0001), but not in chromosomes lacking copy number changes (Fig. 3d). These data indicate that chromosomal imbalances, rather than mutant p53 expression, significantly correlate with transcriptional changes in our isogenic models.

### Clonal in vitro gain-of-function phenotypes are associated with aneuploidy and not mutant p53 expression

**Fig. 4:**
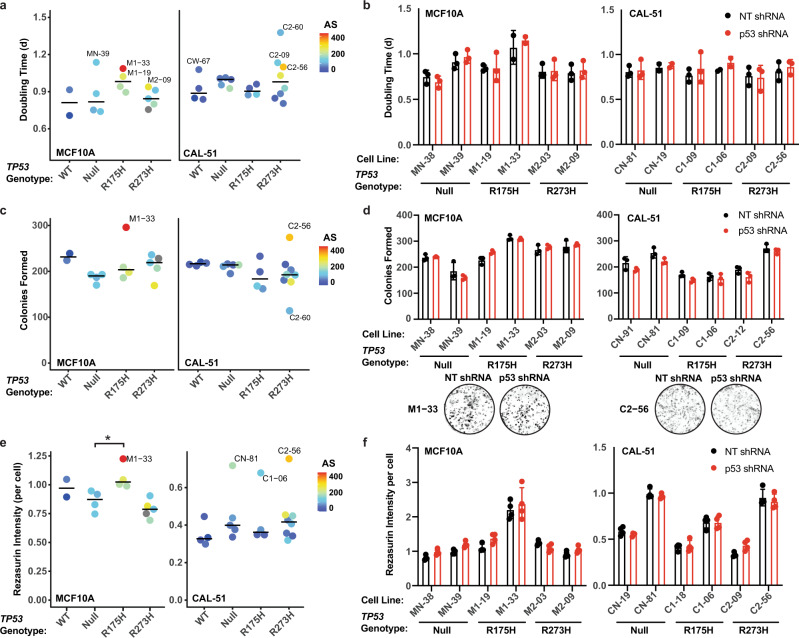
Differences in proliferation, colony formation, and metabolism are associated with aneuploidy and not mutant p53 expression. **a** Average doubling time in all MCF10A (left) and CAL-51 (right) clonal cell lines by *TP53* genotype. **b** Doubling time in MCF10A (left) and CAL-51 (right) cell lines of the indicated *TP53* genotype expressing either a nontargeting (NT, black) or p53 targeting shRNA (red). Mean ± standard deviation (s.d.) from *n* = 3 independent experiments per cell line (except M1-33, CN-19, and C1-06 cell lines, *n* = 2). **c** Average number of colonies formed in all MCF10A (left) and CAL-51 (right) cell lines by *TP53* genotype. **d** Average number of colonies formed in MCF10A (left) and CAL-51 (right) cell lines of the indicated *TP53* genotype expressing either a nontargeting (NT, black) or p53 targeting shRNA (red). Bottom, representative images. Mean ± s.d. of *n* = 3 technical replicates representative of at least two independent experiments per cell line. **e** Resazurin intensity per cell, in all MCF10A (left) and CAL-51 (right) cell lines by *TP53* genotype. **f** Resazurin intensity per cell in MCF10A (left) and CAL-51 (right) nontargeting (NT, black) or p53 shRNA (red) containing cell lines. Mean ± s.d. of *n* = 4 technical replicates, representative of at least two independent experiments per cell line. **a, c, e** Dots represent the mean per cell line (MCF10A *n* = 2 WT, 4 Null, 4 R175H, and 5 R273H cell lines; CAL-51 *n* = 4 WT, 5 Null, 4 R175H, and 8 R273H cell lines) from at least two independent experiments. Bars indicate the median per genotype. Dots are colored by their calculated aneuploidy score (AS), and those colored in gray were not profiled in cytogenomic microarray experiments. One-way ANOVA with Dunnett’s multiple comparison test, *P < 0.026. **b, d, f** Significance tested using two-way analysis of variance with Sidak’s multiple comparisons test. Western blots showing knockdown of p53 are shown in Supplementary Fig. 7a, b. Source data are provided in the Source Data File.

Cancer cells with mutant p53 have been reported to have neomorphic activities leading to oncogenic phenotypes such as increased cellular proliferation rates^6^, increased colony formation^7,8^, altered cellular and mitochondrial metabolism^7,13,44,45^, and chemotherapeutic resistance^46–48^. To determine if our mutant p53 cell lines displayed evidence of GOF activities in vitro, we performed a variety of functional assays using our full panel of MCF10A and CAL-51 cell lines. Assessment of proliferation through cell growth assays revealed no significant differences in doubling time for MCF10A and CAL-51 cells by *TP53* genotype (Fig. 4a) or upon stable p53 knockdown (Fig. 4b, Supplementary Fig. 7a, b). Decreased proliferation has been associated with aneuploid states^49^. Similarly, in both MCF10A and CAL-51 models, the average doubling time for aneuploid-high cells was higher than that of aneuploid-low cells (MCF10A *P* = 0.035, CAL-51 *P* = 0.047) (Supplementary Fig. 8a). Colony-formation assays also revealed no significant differences for MCF10A and CAL-51 cells by *TP53* genotype or with p53 knockdown; however, cell lines with the most colonies formed were among those with the highest AS (M1-33, C2-56, Fig. 4c, d), indicating that changes in cell growth in our clonal cell lines can be attributed to genetic variation caused by aneuploidy rather than *TP53* genotype.

We evaluated our cell lines for changes in cellular metabolism but observed no significant differences in the level of reactive oxygen species (ROS), mitochondrial superoxides, mitochondrial mass, or mitochondrial membrane potential in MCF10A cells by *TP53* genotype (Supplementary Fig. 8b-e). We did observe a significant difference in mitochondrial metabolism when measuring the reduction of resazurin in MCF10A R175H clonal lines; however, this increase was most notable in the cell line with the highest AS (M1-33) and did not change upon p53 knockdown (Fig. 4e, f, Supplementary Fig. 7a). In the CAL-51 lines, there was not a significant difference in resazurin reduction relative to *TP53* genotypes or upon p53 knockdown (Fig. 4e, f and Supplementary Fig. 7b). However, similar to the MCF10A model, cell lines with the highest metabolic activity were among those with the highest AS (CN-81, C1-06, and C2-56, Fig. 2a), and we observed a significant correlation between this metabolic activity and the AS calculated for each clone (*P* = 0.0001, Supplementary Fig. 8f). In CAL-51 R273H mutant cells with varying aneuploidy (Supplementary Fig. 8g), we observed that mitochondrial membrane potential was increased in three aneuploid R273H mutant lines compared to three non-aneuploid R273H mutant lines (Supplementary Fig. 8h). Previous reports have shown that mutant p53 upregulates the mevalonate pathway^12^; however, analysis of RNA-seq data for mevalonate pathway gene expression revealed that expression was clonal, and not associated with the *TP53* genotype or AS, except in the CAL-51 model in which highly aneuploid samples showed increased expression of the gene NAD(P) Dependent Steroid Dehydrogenase-Like (*NSDHL)* (Supplementary Fig. 8i). These data indicate that altered cellular metabolism is associated with aneuploidy and independent of *TP53* genotype.

To determine if R175H or R273H *TP53* mutations confer resistance to commonly used cancer chemotherapeutic agents, we treated all clonal lines in both models with increasing concentrations of doxorubicin or paclitaxel for 72 h. Significant changes in drug sensitivity were observed when comparing cells with p53 LOF (null, R175H, or R273H) to WT cells (MCF10A, doxorubicin [*P* = 0.027]; CAL-51, doxorubicin [*P* = 0.009], and paclitaxel [*P* < 0.0001]) (Supplementary Fig. 9a, b). However, there was no significant difference in sensitivity when comparing either R175H or R273H mutant lines to *TP53* null cells in either model or with knockdown of p53 (Supplementary Fig. 9a-d, Supplementary Fig. 7a, b). The heat shock protein 90 (Hsp90) inhibitor 17-AAG and histone deacetylase (HDAC) inhibitor SAHA (Vorinostat) have been reported to decrease cell viability in mutant p53-containing cancer cells^50,51^. However, CAL-51 cells showed no significant differences in 17-AAG or SAHA IC_50_ values by *TP53* genotype (Supplementary Fig. 9e, f) or after p53 knockdown (Supplementary Fig. 9g, Supplementary Fig. 7b). Compared to aneuploid-low cells, aneuploid-high cells had significantly increased sensitivity to SAHA (*P* = 0.0006) (Supplementary Fig. 9h). Altogether, these data indicate that in vitro GOF phenotypes are not associated with mutant p53 expression but can be associated with genomic alterations such as aneuploidy that occur following the loss of p53 function.

### Clonal differences in tumorigenicity are associated with aneuploidy and not p53 genotype

**Fig. 5:**
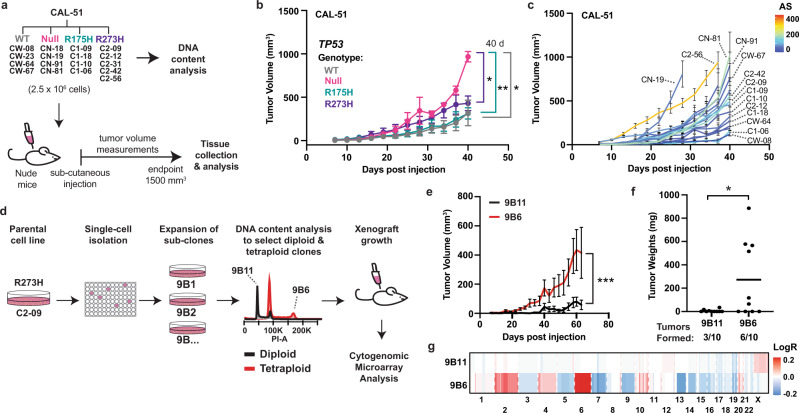
Clonal differences in tumorigenicity are associated with aneuploidy and not mutant p53 expression. **a** Diagram demonstrating workflow for CAL-51 xenograft tumor growth experiment. **b**, **c** Tumor volume (in cubic millimeters) of CAL-51 cell lines. Data shown represent the mean ± standard error of the mean (s.e.m.) tumor volume (*n* = 10 tumors per cell line) measured across CAL-51 cell lines indicated in **a** averaged by **b** *TP53* genotype or **c** colored by aneuploidy score (AS). **d** Diagram demonstrating workflow for single-cell isolation and subcutaneous xenograft experiment of CAL-51 diploid (9B11) and tetraploid (9B6) R273H mutant p53 subclones. **e**, **f** Tumor volume (**e**) and tumor weights (**f**) for 9B11 and 9B6 subclone xenografts. Data shown represent the mean ± s.e.m. tumor volume measured every three days (*n* = 10 tumors per cell line). **g** Copy number alterations from cytogenomic microarray analyses in 9B11 and 9B6 subclones (Log_2_ ratios, LogR). Chromosomal gain (red) and loss (blue). Significance tested using **b** mixed-effects analysis with Dunnett’s multiple comparison test, **e**, **f** Two-tailed Student’s *t*-tests. *P < 0.05, **P < 0.01, ***P < 0.001. Source data and exact *P* values are provided in the Source Data File.

It has been reported that tumor cells with *TP53* mutations display more aggressive features in murine models^4,8^. To investigate if our p53 mutant-containing cell lines displayed gain-of-function activities that would lead to increased tumorigenesis in vivo, we evaluated xenograft tumor growth of our MCF10A and CAL-51 cell lines after subcutaneous injection (Fig. 5a and Supplementary Fig. 10a). MCF10A cell lines did not form tumors in mice, regardless of *TP53* genotype or DNA content (Supplementary Fig. 10b, c). CAL-51 cell lines showed significant differences in tumor growth by *TP53* genotype, with *TP53* null cell lines having significantly increased tumor growth compared to *TP53* WT, R175H, or R273H mutant cells (WT vs Null, *P* = 0.035; R175H vs Null, *P* = 0.008; R273H vs Null, *P* = 0.022; Fig. 5b). While tumor growth was variable across CAL-51 cells regardless of *TP53* genotype, we noticed a trend where CAL-51 cells with a higher AS displayed increased tumor growth (e.g., C2-56, CN-81, C2-42, Fig. 5c, Supplementary Fig. 10d). However, not all highly aneuploid cells showed increased growth (e.g., C1-06). Our data are consistent with previous reports that aneuploidy can be either tumor-promoting or tumor-suppressive^52,53^. Additionally, cell line CN-19 displayed no detectable aneuploidy but was the most tumorigenic (Fig. 5c, Supplementary Fig. 10d). To further investigate the observed correlation between tumor growth and aneuploidy, we isolated a diploid and tetraploid subclonal cell line from C2-09, a CAL-51 R273H mutant cell line with an intermediate growth phenotype (Fig. 5d). The tetraploid clone 9B6 displayed significantly increased xenograft tumor growth (*P* = 0.001, Fig. 5e) and final tumor weight (*P* = 0.019, Fig. 5f) compared to the diploid clone 9B11. Analysis of tumor DNA through CMA revealed the 9B6 clone was highly aneuploid compared to clone 9B11 (Fig. 5g). In summary, cell lines with mutant p53 did not preferentially show increased tumorigenicity in vivo; rather, the feature of increased aneuploidy in p53 mutant cells was associated with increased tumorigenicity in vivo.

### Metastatic phenotypes are associated with aneuploidy and not mutant p53 expression

**Fig. 6:**
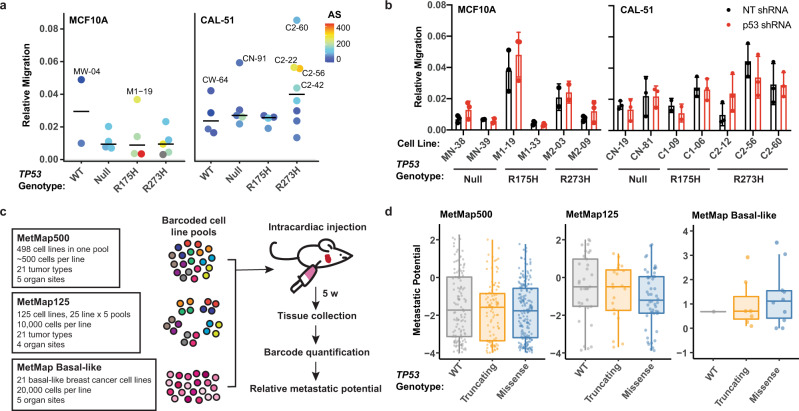
Metastatic phenotypes are associated with aneuploidy and not mutant p53 expression. **a** Quantification of the relative migration (mean cells per field from three technical replicates normalized to the number of cells seeded, arbitrary units) of MCF10A (left) and CAL-51 (right) cells that crossed the membrane in transwell assays. Each dot represents the mean relative migration for each cell line (MCF10A *n* = 2 WT, 4 Null, 4 R175H, and 5 R273H cell lines; CAL-51 *n* = 4 WT, 5 Null, 4 R175H, and 8 R273H cell lines) from at least two independent experiments. Bars represent the median value per *TP53* genotypes. Dots are colored by aneuploidy score, and those colored in gray were not profiled in cytogenomic microarray experiments. **b** Quantification of relative migration, as described above, in MCF10A (left) and CAL-51 (right) cells containing nontargeting (NT, black) or p53 shRNAs (red). Mean ± s.d. from *n* = 3 independent experiments (except MN-39, M2-03, and C1-09 cell lines, *n* = 2). Western blots showing knockdown of p53 are shown in Supplementary Fig. 7b, c. **c** Diagram demonstrating workflow for metastatic datasets generated in the MetMap project^55^. **d** Box and whisker plots of the metastatic potential in cells with wild-type (WT), missense or truncating mutations in *TP53* in the MetMap500 (left; WT *n* = 129, Truncating *N* = 101, Missense *N* = 195), MetMap125 (middle; WT *n* = 33, Truncating *N* = 20, Missense *N* = 50) and MetMap Basal-like (right; WT *n* = 1, Truncating *N* = 7, Missense *N* = 10) datasets. Points represent mean metastatic potential across all sites. Boxplot elements: center line, median; box limits, upper and lower quartiles; whiskers, 1.5× interquartile range. Significance tested using **a** one-way analysis of variance (ANOVA) with Dunnett’s multiple comparisons test, **d** two-way ANOVA with Sidak’s multiple comparisons test, and **c** one-way ANOVA with Tukey’s multiple comparison test. Source data are provided in the Source Data File.

Mutant p53 has been associated with cellular features leading to metastatic progression, such as enhanced migration^6,16–19,54^. We performed transwell migration assays to determine if mutant p53 alters cellular migration in our isogenic cell line models. We did not observe a statistical difference in the average relative migration in either MCF10A or CAL-51 clonally derived cell lines across *TP53* genotypes (Fig. 6a), and knockdown of p53 did not significantly alter migration relative to any genotype in MCF10A or CAL-51 cells (Fig. 6b, Supplementary Fig. 7a, b). While the level of migration was variable across each clonal cell line, the CAL-51 R273H mutant lines with the highest overall relative migration were among those with the most elevated AS (Fig. 6a, clones C2-60, C2-56, and C2-22). Note that not all aneuploid cell lines showed increased migration (e.g., MCF10A M1-33, CAL-51 CN-81, and C1-06), suggesting specific chromosomal alterations and not the degree of aneuploidy resulted in increased migration.

To determine if cells with *TP53* mutations have increased metastasis in vivo, we utilized the MetMap500, MetMap125, and MetMap Basal-like datasets from the metastasis map (MetMap) project, which provided the metastatic potential of barcoded and pooled cancer cell lines following cardiac injection in mice (Fig. 6c)^55^. We compared the metastatic potential of cell lines that were p53 WT or contained either missense or truncating mutations in *TP53*. Across all three datasets, we found no statistical difference in the metastatic potential of cell lines based on *TP53* genotype (Fig. 6d). While there is no correlation between total aneuploidy and metastatic potential in these datasets^55^, others have shown that chromosomal alterations are enriched and drive metastatic tumor formation^52,56^. Similarly, our results indicate that differences in metastatic phenotypes such as increased migration are not dependent on the expression of mutant p53 or total levels of aneuploidy but are likely the result of specific chromosomal alterations.

### Aneuploidy and loss of p53 function associate with unfavorable prognosis

**Fig. 7:**
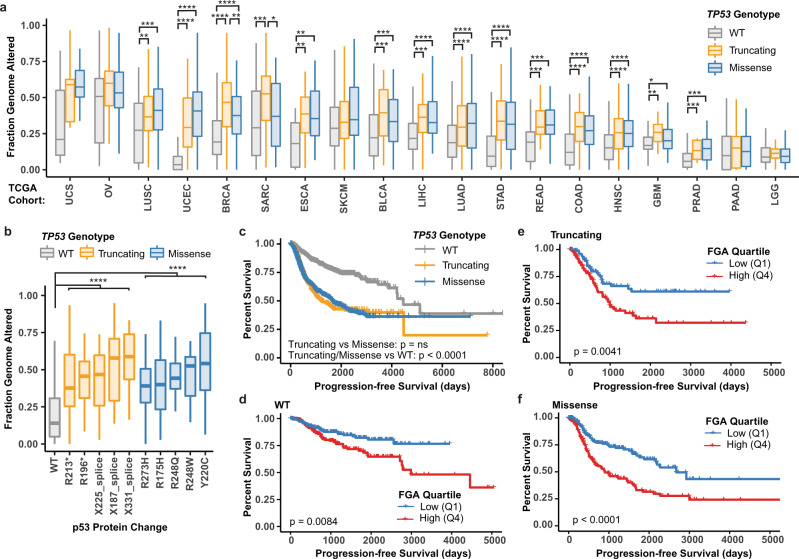
Aneuploidy and loss of p53 function associate with unfavorable prognosis. **a**, **b** Box and whisker plots of the fraction of the genome altered (FGA) in tumors with wild-type (WT), missense or truncating mutations in *TP53* compared **a** across cancer types (source: The Cancer Genome Atlas [TCGA]), or **b** in BRCA, OV, UCEC, UCS, and LUSC cancer types across the five most frequent truncating or missense mutations. Cohort acronyms, values for *n*, and exact *P* values can be found in Supplementary Table 3 or the Source Data file. Pairwise two-sided Wilcoxon test with Benjamini–Hochberg *P*-value correction, *P < 0.05, **P < 0.01, ***P < 0.001, ****P < 0.0001. Boxplot elements: center line, median; box limits, upper and lower quartiles; whiskers, 1.5× interquartile range. **c**–**f** Kaplan–Meier curves showing progression-free survival of individuals with BRCA, OV, UCEC, UCS, and LUSC separated by *TP53* genotype (**c**) or further divided into aneuploid-low (blue, lower quantile FGA, Q1) or aneuploid-high (red, upper quantile FGA, Q4) groups in individuals with tumors containing WT (**d**), truncating (**e**), or missense (**f**) mutant *TP53*. Log-rank tests. Source data are provided in the Source Data File.

*TP53* mutation strongly correlates with the development of aneuploidy^20,21^, and both *TP53* mutation and aneuploidy have been associated with unfavorable prognostic features in multiple cancer types^57–62^. Thus, we determined the relationship between missense *TP53* mutations, aneuploidy status, and survival across human tumor types. We first analyzed the fraction of the genome altered (FGA) by aneuploidy in tumors across 19 cancer types (TCGA, *n* = 6682; abbreviations defined in Supplementary Table 3) that contained either missense or truncating mutations in *TP53*. Similar to previous findings^58^, the FGA was significantly increased across a majority of tumor types with either truncating or missense mutations in *TP53* compared to those with WT p53 (Fig. 7a). Uterine carcinosarcoma (UCS), uterine corpus endometrial carcinoma (UCEC), lung squamous cell carcinoma (LUSC), ovarian (OV), and breast cancers (BRCA) with missense *TP53* mutations displayed the highest median FGA across all cancer types (Fig. 7a). Breast and sarcomas were the only cancer types that displayed a significant difference between missense and truncating *TP53* mutations, with tumors containing truncating *TP53* mutations having increased FGA. Not all missense mutations in *TP53* equally disrupt p53 function, so we assessed aneuploidy in the top five most frequent missense and truncating amino acid changes across the above tumor types with the highest median FGA (UCS, UCEC, OV, BRCA, and LUSC). Tumors with WT p53 had a significantly lower FGA compared to truncating or missense mutations (*P* < 0.0001); however, there was no statistical difference between high-frequency missense and truncating p53 alterations (Fig. 7b). This data suggest that loss of p53 function leads to increased aneuploidy, irrespective of the type of point mutation.

To determine if individuals with tumors containing missense versus truncating mutations experienced differential survival, we compared the progression-free survival in individuals with BRCA, OV, UCEC, UCS, and LUSC. Individuals with tumors containing either missense or truncating mutations in *TP53* displayed significantly worse survival when compared to those with WT p53 (*P* < 0.0001). There was no statistical difference in survival between individuals with tumors containing either missense or truncating mutations in p53 (Fig. 7c). To assess the relationship of tumor aneuploidy with survival, independent from *TP53* mutation, we stratified individuals with tumors containing WT, missense, or truncating mutations in *TP53* into either aneuploid-high (upper quantile FGA) or aneuploid-low (lower quantile FGA) groups. All individuals with aneuploid-high tumors, regardless of p53 genotype, showed significantly worse survival outcomes compared to aneuploid-low tumors (WT *P* = 0.008, truncating *P* = 0.004, missense *P* < 0.0001) (Fig. 7d-f). In summary, these data indicate that progression-free survival is not directly associated with *TP53* mutation type (truncating vs. missense). Instead, an individual’s outcome is associated with the loss of p53 function and increased genomic alterations in their tumor(s).

## Discussion

The concept of mutant p53 GOF was introduced over 30 years ago^4,63^. Since then, many publications have reported context-specific and conflicting evidence for oncogenic phenotypes arising from overexpression of the mutant protein. Evidence supporting the mutant p53 GOF hypothesis includes the accumulation of specific high-frequency hotspot p53 mutants, suggesting that GOF activities confer a fitness advantage. However, many hotspot mutations, including R273 and R175, contain methylated CpG dinucleotides, rendering them more likely to mutate by spontaneous deamination^64^. Recent findings confirmed that natural mutational processes combined with LOF and dominant-negative activities select for the spectrum of *TP53* mutations^65^; additionally, this work found that the growth of an extensive panel of cell lines was not dependent on mutant p53 protein expression. These findings were corroborated by a study of mutant p53 isogenic models of myeloid malignancies showing no evidence of GOF, but instead LOF and dominant-negative activities over WT p53 protein^27^. Two concurrent studies of mouse models harboring the knockin *TP53* mutations equivalent to R175H and R273H showed persuasive but conflicting evidence for GOF in vivo, with an increased incidence of carcinomas in R175H/−^19^ but not R175H/R175H^6^ mice. The latter inconsistency was attributed to differing murine genetic backgrounds. Of note, tumors from R175H/− mice displaying altered tumor spectra also showed enlarged nuclei and polyploid cells in hematoxylin and eosin-stained tumor sections^19^, consistent with the notion that aneuploidy could underlie differences in tumor development and GOF activities reported using these models.

The increased aneuploidy observed in our mutant p53 cell lines is consistent with previous studies^37,66^. There is a strong correlation between *TP53* mutation and aneuploidy in human tumors^20,21^, and analyses of medulloblastoma tumors and acute myeloid leukemias from Li-Fraumeni Syndrome (LFS) individuals with somatic *TP53* mutations showed increased chromothripsis^67^. Fibroblasts from LFS individuals accumulate aneuploid cells^68,69^, as do normal human and murine fibroblasts with exogenous expression of *TP53* missense mutations^9,70,71^. Further, a study of LFS individuals revealed that those who developed cancer had a striking enrichment in germline copy number variation^72^, suggesting that aneuploidy following p53 LOF leads to tumorigenesis. The latter is supported by the wide range of tumorigenic phenotypes caused by aneuploidy^23,25,26^, many of which have been associated with mutant p53 GOF activities, including, but not limited to: altered proliferation^49^, altered metabolism^73,74^, transcriptional reprogramming and drug resistance^75^, immune evasion^22^, migration^52^, and invasion and metastasis^56^. Given the strong propensity for cells containing mutant p53 to become aneuploid and the overlap of tumorigenic phenotypes related to both alterations, GOF phenotypes identified in mutant p53 models must be carefully validated relative to corresponding chromosomal changes.

The underlying mechanisms behind the increased aneuploidy we observed in our engineered lines require further study, although many potential mechanisms, such as loss of cell cycle checkpoints, have been previously reported^37^. Our study was limited by the number of independent cell lines assayed and the technology available to evaluate aneuploidy. Finally, our work only examined two specific hotspot p53 missense mutations in two cell line models; thus, we cannot exclude the existence of select GOF activities occurring in cells containing other p53 missense mutants or existing in different tissue-specific cellular contexts. In addition, further studies are needed to determine the effect of oncogenic mutations, not present in the model systems used in the current study, on potential mutant p53 GOF activities.

In summary, our study demonstrates that the acquisition of aneuploidy can generate a variety of the previously ascribed mutant p53 GOF phenotypes. Further, our data showed the heterogeneity of genomic alterations that can occur following mutation of p53, consistent with the diverse and sometimes conflicting phenotypes observed in prior reports. While we cannot rule out the existence of p53 mutant-specific GOF effects in other models, future studies should carefully consider how the genomic changes that occur after the loss of WT p53 can confound and contribute to GOF phenotypes. Acquisition of aneuploidy after the loss of WT p53 function provides a unifying mechanism that accounts for the wide array and context-specific nature of GOF phenotypes previously attributed to p53 mutant proteins.

## Methods

### Cell culture

The CAL-51, CAL-51 isogenic clonal cell lines, and 293FT cells were cultured in DMEM with 10% (v/v) FBS. The MCF10A cell line and isogenic clonal cell lines were cultured in DMEM:F12 with 5% horse serum, 100 ng/mL cholera toxin, 500 ng/mL hydrocortisone, 20 ng/mL human epidermal growth factor, and 10 μg/mL insulin. All cell lines were maintained in the described media with 1% penicillin and streptomycin in a 37 °C incubator at 5% carbon dioxide. Cells were routinely tested to be negative for mycoplasma. DNA fingerprinting analysis was performed on CAL-51 and several isogenic clones in March 2016. MCF10A and CAL-51 parental isogenic clonal cell lines were also validated against known karyotypes (compare Supplementary Fig. 2a and Fig. 2a).

### CRISPR/Cas-mediated genome editing

Genome editing using the CRISPR/Cas9 system was performed in close adherence to the Zhang lab’s protocol^32^. Guide RNAs (gRNAs) were designed in the Benchling web tool (https://www.benchling.com/); 20-nucleotide (nt) gRNAs were selected (3–5 per desired mutation site) with the highest target specificity score that cut within at least 15nt of the desired mutant base for screening. Complementary guide oligos were ordered with an initial 5’ guanine (if not already present) and BbsI overhangs for cloning into pSpCas9(BB)-2A-GFP (PX458) (Addgene #48138). Guide oligos were resuspended at 100 μM, phosphorylated, and annealed through incubation with T4 PNK and ligation buffer. Cloning into PX458 was conducted by incubating the plasmid and diluted oligo duplexes with Tango buffer, DTT, ATP, FastDigest BbsI, and T7 ligase. The ligation products were subsequently treated with PlasmidSafe ATP-dependent DNase to digest residual linear DNA. Cloned plasmids were transformed into DH5α competent cells and selected on LB agar plates containing 100 μg/mL ampicillin. Plasmid DNA was isolated from the resulting colonies using a QIAprep Spin Miniprep Kit (Qiagen), and sequence verified by Sanger sequencing.

The following was performed to assess gRNA activity. Cloned plasmids were transfected into 293FT cells using Lipofectamine 3000; and, 72 h later, DNA was isolated from the transfected cells using QuickExtract DNA extraction solution (Epicentre) per manufacturer’s recommendations. Genomic DNA was amplified with primers designed to generate an asymmetric ~500 bp PCR product around the gRNA cut site (Supplementary Table 4) using Platinum Taq DNA Polymerase High Fidelity. PCR products were purified using a QIAQuick PCR Purification Kit (Qiagen) and annealed by a gradual reduction in temperature from 95 °C, followed by SURVEYOR digestion using SURVEYOR nuclease S and enhancer S with supplemented magnesium chloride. SURVEYOR digestion products were run on a 5% polyacrylamide TBE gel and visualized using SYBR Gold. gRNAs producing a prominent asymmetric digestion product were selected for further use (R175H & null (amino acid position 181): CACCGTATCTGAGCAGCGCTCATGG; null (amino acid position 196): CACCGTCCTCAGCATCTTATCCGAG; R273H: CACCGTGCGTGTTTGTGCCTGTCC). A negative control (no transfected plasmid) was included to rule out digestion products generated by endogenous single-nucleotide polymorphism (SNP) mismatches.

Once a gRNA was selected for a specific mutant site, homology-directed repair (HDR) templates were designed using the Benchling web tool. HDR templates were designed to contain mutant bases corresponding to clinically observed hotspot mutations, along with a synonymous mutation in the protospacer adjacent motif (PAM) to prevent CRISPR/Cas9-mediated cleavage of the HDR-recombined alleles and ~75 bp of flanking homologous sequence on each side. For the following HDR templates, nucleotides surrounded by brackets indicate the clinically observed missense mutation, and lower-case nucleotides depict synonymous mutations designed to disrupt the PAM site or reduce guide RNA complementarity.

R175H:CACCCCCGCCCGGCACCCGCGTCCGCGCCATGGCCATCTACAAGCAGTCACAGCACATGACGGAGGTTGTGAGGC[A]CTGCCCaCACCATGAGCGCTGCTCAGATAGCGATGGTGAGCAGCTGGGGCTGGAGAGACGACAGGGCTGGTTGCC;

R273H:TCCTTACTGCCTCTTGCTTCTCTTTTCCTATCCTGAGTAGTGGTAATCTACTGGGACGGAACAGCTTTGAGGTGC[A]TGTTTGcGCtTGTCCTGGGAGAGACCGGCGCACAGAGGAAGAGAATCTCCGCAAGAAAGGGGAGCCTCACCACGA.

When the engineering of a synonymous PAM mutant was not possible due to codon position, two additional synonymous mutations were engineered into the gRNA target region to reduce complementarity. HDR templates were ordered as Ultramer oligos (IDT).

CRISPR/Cas9-mediated editing of cells was conducted by incubating 2.5 μg of cloned PX458 plasmid, 5 μL of P3000 reagent, 7.5 μL of Lipofectamine 3000, and if attempting knockin mutagenesis rather than frameshift knockout, an additional 5 μL of 10 μM single-stranded HDR template in 250 μL of Opti-MEM. The resulting DNA-lipid complexes were added to one well of a subconfluent six-well plate, with the number of wells scaled as necessary. After 48 h, transfected cells were trypsinized, washed in PBS, resuspended in 4% (v/v) FBS in PBS, and stained with 0.5 μg/mL propidium iodide for live/dead discrimination. GFP-positive live cells were gated using lipofectamine-only negative control cells with and without propidium iodide along with transfected cells lacking propidium iodide. Fluorescence-activated cell sorting was performed by the Vanderbilt Flow Cytometry Shared Resource. GFP+/PI− cells were sorted into 96-well plates containing complete culture medium for isolation of single-cell clones.

Clonal populations were expanded 21–28 d after sorting, and DNA was isolated using QuickExtract DNA extraction solution (Epicentre) to determine the *TP53* genotype. Genomic DNA was amplified with primers designed to generate an asymmetric 500 bp PCR product around the gRNA cut site using Platinum Taq DNA Polymerase High Fidelity. PCR products were purified using a QIAQuick PCR Purification Kit (Qiagen) and screened using restriction enzyme cut sites present only in HDR-recombined sequences (BtsI for R175H, NlaIII for R273H). Cells that passed the restriction digest check were further verified by Sanger sequencing. When mixed sequencing traces were present due to heterozygous frameshift alleles, allele cloning was conducted by amplifying genomic DNA using primers described above with EcoRI or BamHI restriction site overhangs, followed by digestion and cloning into the pUC19 vector (New England BioLabs) for transformation. Once bacterial colonies were selected and expanded, DNA isolation was performed using a QIAprep Spin Miniprep Kit (Qiagen), and individual clones were Sanger sequenced to identify single trace alleles. All clonal cell line genotypes were further validated through the analysis of variants from whole-exome sequencing and RNA sequencing (see details below).

### Xenograft tumor studies

Mice were housed and treated in accordance with NIH guidelines and protocols approved by the Institutional Animal Care and Use Committee at the Vanderbilt University Medical Center. Mice were maintained on a 12 h light-dark cycle at 20–26 °C and 30–70% humidity, and housed in ventilated cages with constant access to food and water. For cell line xenograft studies, female 6-8 week-old athymic nude mice (The Jackson Laboratory, #002019) were anesthetized using isoflurane and injected subcutaneously in the left and right flank with 5 × 10^6^ MCF10A or 2.5 × 10^6^ CAL-51 cells suspended in 200 µL PBS. Mice were monitored weekly and palpable tumors measured with digital calipers every 3 days until the experimental endpoint, at which time mice were euthanized by isoflurane overdose and cervical dislocation. Tumor volumes were calculated as width^2^ × length/2. Replicate mice injected with the same cell line were removed from the study when a single mouse had a tumor volume exceeding 1500 mm^3^. Tumors used for subsequent molecular analysis were flash-frozen in liquid nitrogen and stored at −80 °C. All analyses were performed blinded to *TP53* genotype or aneuploidy status.

### shRNA-mediated gene knockdown

*TP53* (Mission TRCN0000003753 or TRCN0000342259) or nontargeting negative control (MISSION pLKO.1-puro Non-Mammalian shRNA Control, SCH002; Mission pLKO.5-puro TRC2 Non-Mammalian, SHC202) shRNAs were purchased from Sigma–Aldrich. Lentivirus was produced by co-transfection of shRNA-expressing viral vectors (10 µg) with packaging plasmids PMD2.G (2.5 µg, Addgene, 12259) and pxPAX2 (7.5 µg; Addgene, 12260) into 293FT cells. Viral supernatant was harvested 48 h after transfection and passed through a 0.45 µm syringe filter to clear cellular debris. Viral aliquots were stored at −80 °C prior to infection and limited to one freeze-thaw cycle before use. Target cells were infected with a 1:10 dilution of the virus in the presence of 10 µg/mL polybrene for 24 h. After fresh media were added, cells were grown for 48 h until antibiotic selection with puromycin (CAL-51, 0.625 µg/mL; MCF10A, 1 µg/mL). Knockdown was verified through immunoblotting and RNA sequencing.

### Immunoblotting

All cells were lysed in RIPA buffer (pH 7.4) buffer (150 mM NaCl, 50 mM Tris-HCl [pH 7.5], 0.1% SDS, 1.0% NP-40, 0.5% Deoxycholic Acid, 5 mM EDTA) supplemented with 1 mM DTT, phosphatase inhibitors (50 mM NaF, 0.2 mM Na_3_VO_4_), and protease inhibitors (10 mg/mL CLAP, 200 mg/mL AEBSF). Protein concentration was determined (Modified Lowry Protein Assay Kit, ThermoFisher), and equal amounts of protein separated on SDS polyacrylamide gels and transferred to PVDF membrane. Western blots were incubated with primary antibodies overnight at 4 °C. Membranes were washed and incubated in HRP-conjugated secondary antibodies (goat anti-Mouse or goat anti-Rabbit, Thermo Fischer Scientific, 31432 and 31462) for 1 h at room temperature. Membranes were again washed and developed using ECL kits and digitally imaged (Amersham Imager 600, GE Healthcare Life Sciences). Western blots were quantified relative to loading controls with Fiji^76^. Primary antibodies used include: p53 DO-1 (1:1000, Santa Cruz, sc-126), GAPDH (1:1000, Merck Millipore, MAB374), actin (1:1000, Santa Cruz, sc-47778), vinculin (1:1000, Invitrogen, 700062), MDM2 (1:200, Santa Cruz, sc-965), and p21 (1:200, Santa Cruz, sc-6246).

### RNA sequencing and geneset enrichment analysis

Cell lines were harvested by scraping, and pellets were frozen at −80 °C. RNA was extracted using ZYMO Quick-RNA MiniPrep Kits (Zymo Research) or with Aurum Total RNA Mini kit for p53 knockdown RNA-seq experiments according to the manufacturer’s instructions. RNA concentration and purity were measured on a NanoDrop spectrophotometer, and 2 μg RNA (A260/280 > 2) were sent for sequencing (VANTAGE core at Vanderbilt University Medical Center or Novogene Co. Ltd., Beijing, China). Sequencing libraries were generated using Illumina TruSeq or NEBnext Ultra RNA Library Prep Kits following the manufacturer’s recommendations. Sample quality control to test for RNA integrity and contamination was performed using Agarose Gel electrophoresis and the Agilent 2100 Bioanalyzer System. Library preparations were sequenced by the Novaseq 6000 platform, generating unstranded 150 bp paired-end reads, and resulting in ~19–30 million reads per sample. Raw RNA-seq reads (FASTQ files) were trimmed to remove adapter sequences using Flexbar v3.4.0^77^, and quality was evaluated using FastQC v0.11.7^78^ and MultiQC v1.5^79^. Reads were quantified against GENCODE v28 transcripts using Salmon v0.12.0^80^ correcting for sequence-specific bias and fragment-level GC bias. Transcript abundances were imported into R v3.6.1 and summarized to the gene level using tximport 1.12.3^81^. DESeq2 v1.24.0^82^ was used for differential gene expression and PCA analysis with independent hypothesis weighting IHW_1.12.0^83^. Shrunken log-fold changes were generated using ashr_2.2-47^84^. Genes were classified as differentially expressed if they had a false discovery rate (FDR) adjusted *p*-value less than 0.1 and a Log2-fold change of ≥1 or ≤−1. Geneset enrichment analysis (GSEA v4.0.3)^85^ was conducted with the MSigDB 7.0 genesets using GSEA Preranked in the default parameters. Genes were ranked according to the Wald statistic output from DESeq2.

### DNA content analysis using flow cytometry

One million cells were prepared for cell cycle analysis by trypsinization and washing with PBS, followed by fixation in 70% ethanol. Cells were stained in a solution consisting of 0.1% (v/v) Triton X-100 and 20 µg/mL propidium iodide. Samples were analyzed at the Vanderbilt Flow Cytometry Shared Resource on a 3-laser LSRSII. Single cells were selected by pulse processing and visualized using FlowJo (10.0.7) (Supplementary Fig. 11).

### DNA content analysis using metaphase spreads

Subconfluent cells (~75%) in a 10 cm culture dish were treated for 1 h with 0.5 μg/mL KaryoMAX colcemid prewarmed at 37 °C. Media were removed and reserved, and cells were trypsinized and resuspended in the reserved media. After centrifugation (300 × *g*, 5 min, 4 °C), cells were gently resuspended with 0.2 mL media and combined with 5 mL of 0.075 M potassium chloride dropwise while vortexing gently. Cells were incubated at 37 °C for 25 min, with gentle inversion every 5 min to keep cells in solution. Cells were then prefixed with 0.5 mL prechilled methanol:glacial acetic acid (3:1) fixative solution dropwise while vortexing gently. Cells were centrifuged (300 × *g*, 5 min, 4 °C) and gently resuspended with 5 mL fixative solution. After storage at 4 °C, cells were dropped onto prechilled Superfrost Plus microscope slides and air-dried at a slant for a minimum of 1 h in the dark. Cells were then mounted with ProLong Gold Antifade Reagent with DAPI and coverslipped. Metaphase spreads were imaged (>15 individual cells per cell line) using fluorescence microscopy with an oil-immersion ×100 objective, and individual chromosomes counted manually using Fiji.

### Cytogenomic microarray and copy number analysis

Whole-genome cytogenomic microarray and copy number analysis were performed at the VUMC Cytogenetics Laboratory using the CytoScan HD SNP microarray platform (Thermo Fisher). Briefly, 250 ng of whole genomic DNA isolated from cultured cell lines was digested with NspI, ligated with NspI adapter primers, and amplified using Platinum Taq with a GeneAmp PCR System 9700. PCR products were purified, fragmented, labeled with biotin, and hybridized to the microarray chip. Chips were washed, stained, and scanned on an Affymetrix scanner. Raw CEL files were analyzed using the Rawcopy R package (http://rawcopy.org)^86^. Segmented log ratios and frequency plots were generated using the copy number R package (v_1.24.0)^87^. Differential chromosome regions were analyzed with CNApp^88^. Aneuploidy scores were generated relative to the WT parental clone and calculated as the sum of the absolute value of the segmented log ratios for each profiled cell line. Cell lines were classified by AS quantile as aneuploid-high (upper quantile, MCF10A AS > 198.5, lines M1-33, M1-19, M1-12, and M2-9; CAL-51 AS ≥ 128.3, lines CN-81, C2-56, C2-22, C2-42, C2-09, and C1-06) or aneuploid-low (lower quantile, MCF10A AS < 114.1, lines MW-04, MW-27, MN-37, and M2-13; CAL-51 AS ≤ 43.7, lines CW-08, CW-23, CW-64, C1-18, C1-10, and C2-38).

### Whole-exome sequencing

Cell lines were harvested by scraping and pellets frozen at −80 °C. Genomic DNA was extracted using Qiagen DNeasy Blood and Tissue kit, according to the manufacturer’s instructions. DNA concentration and purity were measured on a NanoDrop spectrophotometer, and 500 ng were submitted for sequencing at the Vanderbilt Technologies for Advanced Genomics (VANTAGE). Library preparation and capture were performed using the Twist Exome library prep and capture kit (Twist Bioscience). Sequencing was performed at 150 bp Paired-end on Illumina NovaSeq 6000, targeting an average of 20 M reads per sample for 50× coverage. Data analysis was performed using an NGSPERL based custom pipeline^89^. Demultiplexed raw sequencing files were trimmed with Cutadapt (2.10)^90^ and read quality was evaluated using FastQC before and after adapter trimming. All trimmed reads were aligned using BWA (0.7.17-r1188)^91^ to the human genome (v38). The mapped result was refined by local realignment and base quality recalibration using GATK3, and all reads containing soft clip were discarded. Single-nucleotide variants were called using GATK4^92^ with Variant Quality Score Recalibration (VQSR) mode. The SNVs with inbreeding coefficient less than −0.2, a depth less than 10 in any sample, or genotype quality less than 20 in any sample were discarded. SNVs with a minor allele frequency of less than 0.3 in more than 90% of the samples were also discarded. Valid SNVs were annotated by ANNOVAR (20180416)^93^. All SNVs with minor allele frequencies larger than 0.001 in any of ExAC, 1000 G, gnoMad, or TOPMed databases were treated as high-frequency SNVs in population and removed. Copy number variations were also called based on GATK4 germline best practice^92^. Mutation analysis was conducted using the R maftools package^94^.

### Drug sensitivity assays

For adherent viability assays, cells were seeded at 1500 cells per well in quadruplicate in 96-well plates and treated with a six-point, three-fold dose-escalation alongside untreated controls for 72 h. AlamarBlue (ThermoFisher Scientific) was used according to manufacturer recommendations, and fluorescence measured and analyzed with microplate data collection and analysis software Gen5 (Biotek). Viability and IC_50_ were analyzed in Prism (Graphpad, 8.4.3) by performing a non-linear fit to log-transformed normalized fluorescence values.

### Doubling time analysis

For doubling time experiments, cells were seeded at 1500 cells per well in quadruplicate into five 96-well plates. Every 24 h over 5 days, plates were fixed with 100% methanol for 10 min and stored in PBS at 4 °C until imaging. Hoechst 33342 was added to cells (5 µg/µL), and nuclei were counted using the ImageXpress instrument in the Vanderbilt High Throughput Screening core. Nuclei were counted using MetaXpress Multi-Wavelength Cell Scoring Module V6.6.3.733 (Molecular Devices), and doubling time was calculated using the following formula:

$${{{{{\mathrm{Doubling}}}}}}\,{{{{{\mathrm{time}}}}}}=\frac{{{{{{\mathrm{duration}}}}}}\,\ast \,\log (2)}{\log ({{{{{\mathrm{final}}}}}}\,{{{{{\mathrm{concentration}}}}}})-\log({{{{{\mathrm{intial}}}}}}\,{{{{{\mathrm{concentration}}}}}})}$$

### Colony-formation assay

Cell lines were seeded onto 12-well plates (Corning) (MCF10A, 500; CAL-51, 1,000 cells per well) and incubated for seven days. Colonies were fixed with 100% methanol for 10 min and stained with a 1:1 mixture of methanol and crystal violet aqueous solution (Electron Microscopy Sciences) at room temperature for 1 h. Cells were washed three times with dH_2_O before places inverted to dry. Images were taken using the Odyssey infrared imaging system (Li-COR) and colonies counted using CellProfiler^95^.

### Metabolic staining

To measure mitochondrial membrane potential and mass, cells were stained with TMRE and MitoTracker Green (each at 0.2 µM), respectively. Cellular ROS was measured with DCFDA reagent (2.9 µg/mL). Mitochondrial ROS was measured with MitoSOX Red Reagent (3.85 µg/mL). After incubating for 30 min at 37 °C, DCFDA and MitoTracker Green staining were visualized by flow cytometry with the MACSQuant Analyzer 10 FITC channel, while TMRE and MitoSOX staining was visualized with the PE channel (Miltenyi Biotec). Data were analyzed using FlowJo version 10.5.3. For resazurin staining, cells were seeded at 5000 cells per well in quadruplicate in 96-well plates. After 48 h, AlamarBlue was applied at a 1× concentration, and cells were incubated for 6 h. Fluorescence was analyzed with microplate data collection and analysis software Gen5 (Biotek). Cells were then fixed and counted as described for doubling time assay.

### Transwell migration assay

MCF10A (1.5 × 10^5^) or CAL-51 (2 × 10^5^) cells were plated in triplicate onto transwells with 8 µm pore polycarbonate membrane inserts (Corning) with serum-free medium. Complete medium was used as a chemoattractant in the lower chamber. After 18 h, cells were fixed with 100% methanol for 10 min and stained with a 1:1 mix of crystal violet in 100% methanol. Nonmigrated cells on the upper side of the insert were removed with a cotton swab. In parallel, cells were separately plated onto 24-well plates without transwell inserts to determine the total number of attached cells by fixing, imaging, and counting Hoechst 33342 stained nuclei as described for resazurin metabolic assay. The relative migration was calculated as the number of migrated cells normalized to the total number of cells. For each experiment, the number of cells from each image (CAL-51, crystal violet stained ×4 magnification; MCF10A, Hoechst stained, ×20 magnification) was counted using CellProfiler^95^.

### Cancer cell line aneuploidy analysis

Cancer cell line encyclopedia^96^ (CCLE) and *TP53* alteration data were acquired from Memorial Sloan Kettering Cancer Center cBioPortal (http://www.cbioportal.org)^97,98^. MCF10A SNP6 copy number data were acquired from https://www.synapse.org/#!Synapse:syn2346643/wiki/62255^99^. The FGA by aneuploidy was calculated as the length of Log_2_ ratio segments > |0.2|, divided by the length of all segments measured. MCF10A and CAL-51 copy number heatmaps were visualized using the Integrative Genomics Viewer (IGV 2.3.97)^100^.

### Analysis of cancer cell line metastasis

Metastasis map (MetMap)^55^ data were acquired from https://depmap.org/metmap/data/index.html. CCLE annotation and *TP53* mutation data (21Q1) were downloaded from the depmap portal (https://depmap.org/portal/download/). WT *TP53* samples were defined as cell lines that had silent mutations or no mutation in *TP53*. Missense cell lines were defined as those that contained a single *TP53* missense mutation. Cell lines with truncating mutations were defined as those containing nonsense, frameshift, or splice-site mutations in *TP53*.

### TCGA aneuploidy and clinical analysis

TCGA MC3 mutation data (mc3.v0.2.8.CONTROLLED.maf.gz)^101^ were used to determine the *TP53* genotype and was downloaded from the NCI Genomic Data Commons. WT *TP53* samples were defined as individuals that had DNA sequencing data but no mutation calls, silent or noncoding mutations. Samples with multiple alterations or in-frame mutations in *TP53* were removed from the analysis. Missense tumors were defined as those that contained a single *TP53* missense mutation. Tumors with truncating mutations were defined as those containing nonsense, frameshift, stop-gain, or splice-site mutations. The FGA by aneuploidy for TCGA samples was acquired from cBioPortal (http://www.cbioportal.org). Progression-free survival data were obtained from the TCGA Pan-Cancer Clinical Data Resource^102^. TCGA cohorts with low frequency (<10%) or fewer than 20 individuals with *TP53* missense mutations were excluded. Survival curves were constructed with the R survival package^103^ using the Kaplan–Meier method, and the difference between groups was assessed by the Log-rank test. Individuals with multiple tumors were excluded from the analysis. Survival was compared by *TP53* genotype or between individuals with aneuploid-low (lower quantile FGA) and aneuploid-high (upper quantile FGA) tumors with either WT, missense, or truncating *TP53* mutations.

### Statistical analysis and reproducibility

Statistical analyses and visualization were conducted using R (version 3.6.1 or 4.0.0) or Prism (version 8.4.3). All statistical tests were two-sided and statistical details, including measures of centrality, dispersion, and sample size, are indicated in the figures, figure legends and source data. Definitions of statistical significance are defined in the figure legends and results are shown in figures if statistical significance was determined. Analysis between multiple groups was performed using one-way analysis of variance (ANOVA), two-way ANOVA, or through a mixed-effects model, each with multiplicity adjusted *P*-values. Statistical comparison between two groups was performed using Student’s *t*-tests or pairwise Wilcoxon test with Benjamini–Hochberg *p*-value correction. Correlation was analyzed using the Pearson method and visualized with a simple linear regression with 95% confidence bands. Survival curves were constructed using the Kaplan–Meier method, and the Log-rank test assessed the difference between groups.

### Reporting summary

Further information on research design is available in the Nature Research Reporting Summary linked to this article.

## Supplementary information

Supplementary Information

Description of Additional Supplementary Files

Supplementary Data 1

Reporting Summary

## Data Availability

The cytogenomic and processed sequencing data that support the findings of this study are available at https://data.mendeley.com/datasets/vr8fcbczz5/2 [10.17632/vr8fcbczz5.2]. The raw sequencing data that support the findings of this study have been deposited to the Sequence Read Archive under the BioProject accession number PRJNA669391. All other data supporting the findings of this study are available within the article, its supplementary information files and the source data provided with this paper. Publicly available data used in this manuscript were obtained from Public MC3 TCGA MAF, https://gdc.cancer.gov/about-data/publications/mc3-2017; TCGA Survival Data, https://gdc.cancer.gov/about-data/publications/pancanatlas; CCLE, https://portals.broadinstitute.org/ccle and https://depmap.org/portal/download/; MCF10A copy number data, https://www.synapse.org/ -!Synapse:syn2346643/wiki/62255 and MetMap data, https://depmap.org/metmap/data/index.html. Source data are provided with this paper.

## Source data

Source Data

## Code availability

The code used in this study is available from the corresponding author upon reasonable request and at https://github.com/shengqh/TP53_isogenics_exomeseq_3772_hg38.
