# Supplementary material for: Acquisition of aneuploidy drives mutant p53-associated gain-of-function phenotypes
Source: Nat Commun. 2021 Aug 31;12:5184. doi: 10.1038/s41467-021-25359-z (PMC8408227; doi:10.1038/s41467-021-25359-z)
Supplement: Supplementary file 1 — Supplementary Information [file 41467_2021_25359_MOESM1_ESM.pdf]

Acquisition of aneuploidy drives mutant p53-associated gain-of-function phenotypes

Supplementary Information

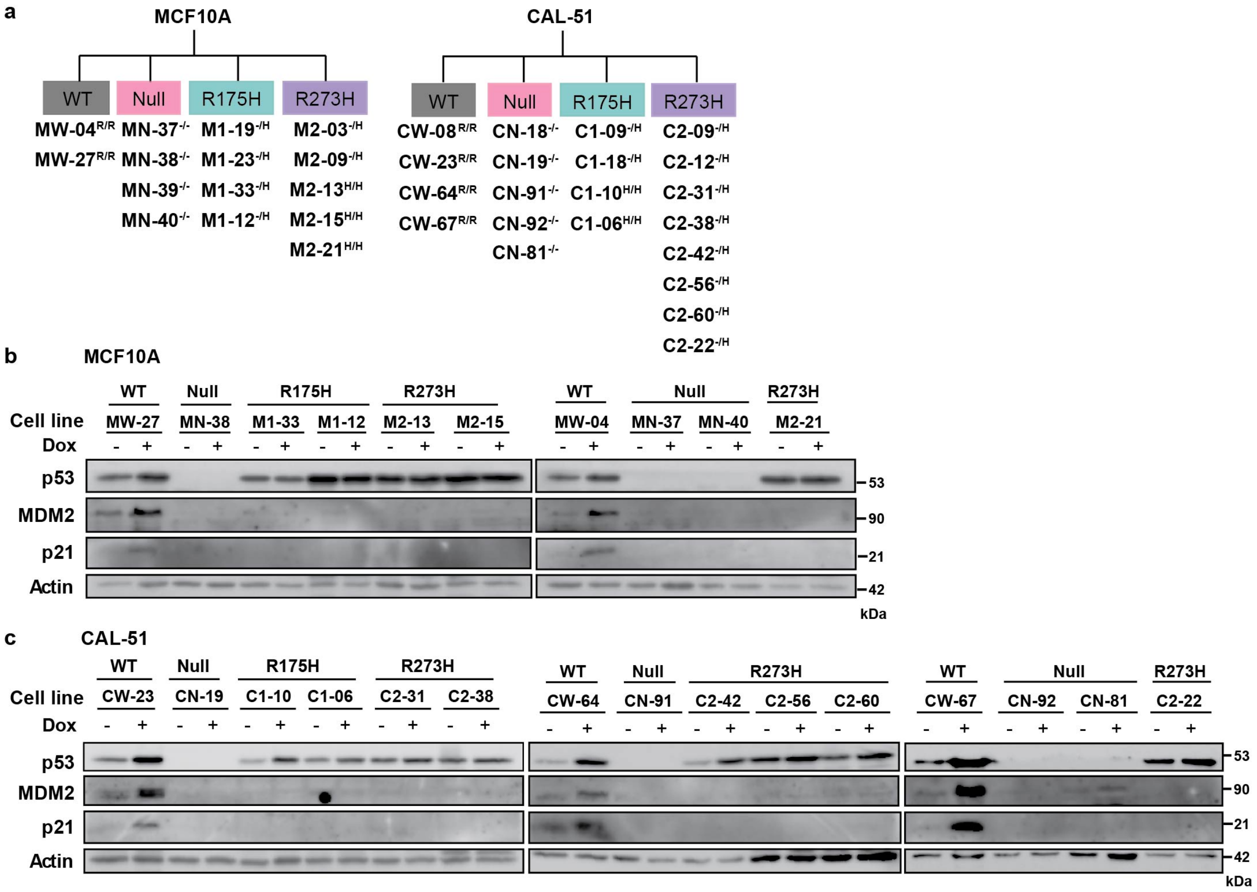

Supplementary Fig. 1. Characterization of genetically-engineered epithelial cell line models to study potential mutant p53 GOF activities.

a Diagram of clonally-derived CRISPR-Cas9 isogenic MCF10A (left) and CAL-51 (right) cell lines grouped by *TP53* genotype (WT, Null, R175H, or R273H). **b, c** Western blots of relative p53, p21, MDM2, and actin protein levels in (**b**) MCF10A and (**c**) CAL-51 clonal cell lines from panel (**a**) treated with and without 0.2 µM doxorubicin for 6 h. Blots are representative of at least two independent experiments. Source data are provided in the Source Data File.

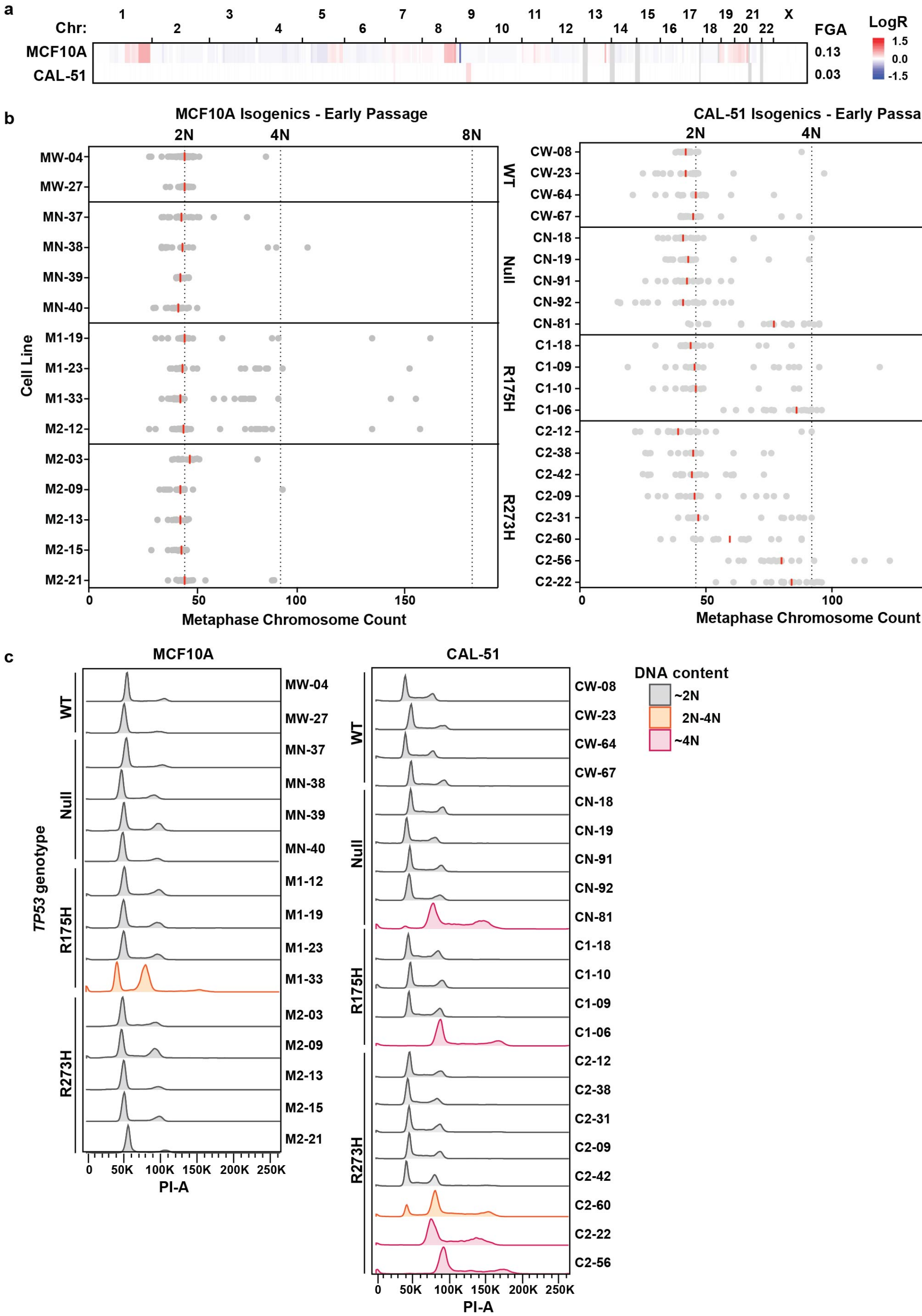

Supplementary Fig. 2. DNA content analysis of MCF10A and CAL-51 early passage isogenic cell lines.

a Heat map of chromosomal alterations (Log$_2$ ratios, LogR), either gain (red) or loss (blue) across the genomes of MCF10A and CAL-51 cell lines and the corresponding fraction of the genome altered

(FGA). Chr, chromosome. **b** Quantification of chromosomes using metaphase spreads (≥15 cells per clonal cell line) for the indicated clonal cell lines in the MCF10A (left) or CAL-51 (right) isogenic models at passage five after clonal expansion. Red bars indicate the median chromosome number. Outliers with >200 chromosomes were removed for visualization. **c** Histograms showing DNA content of MCF10A (left) and CAL-51 (right) isogenic cell line populations by flow cytometry of propidium iodide (PI) stained cells at passage five after clonal expansion. Colors represent DNA content (gray = ~2N, orange = 2N-4N, pink = ~4N). Source data are provided in the Source Data File.

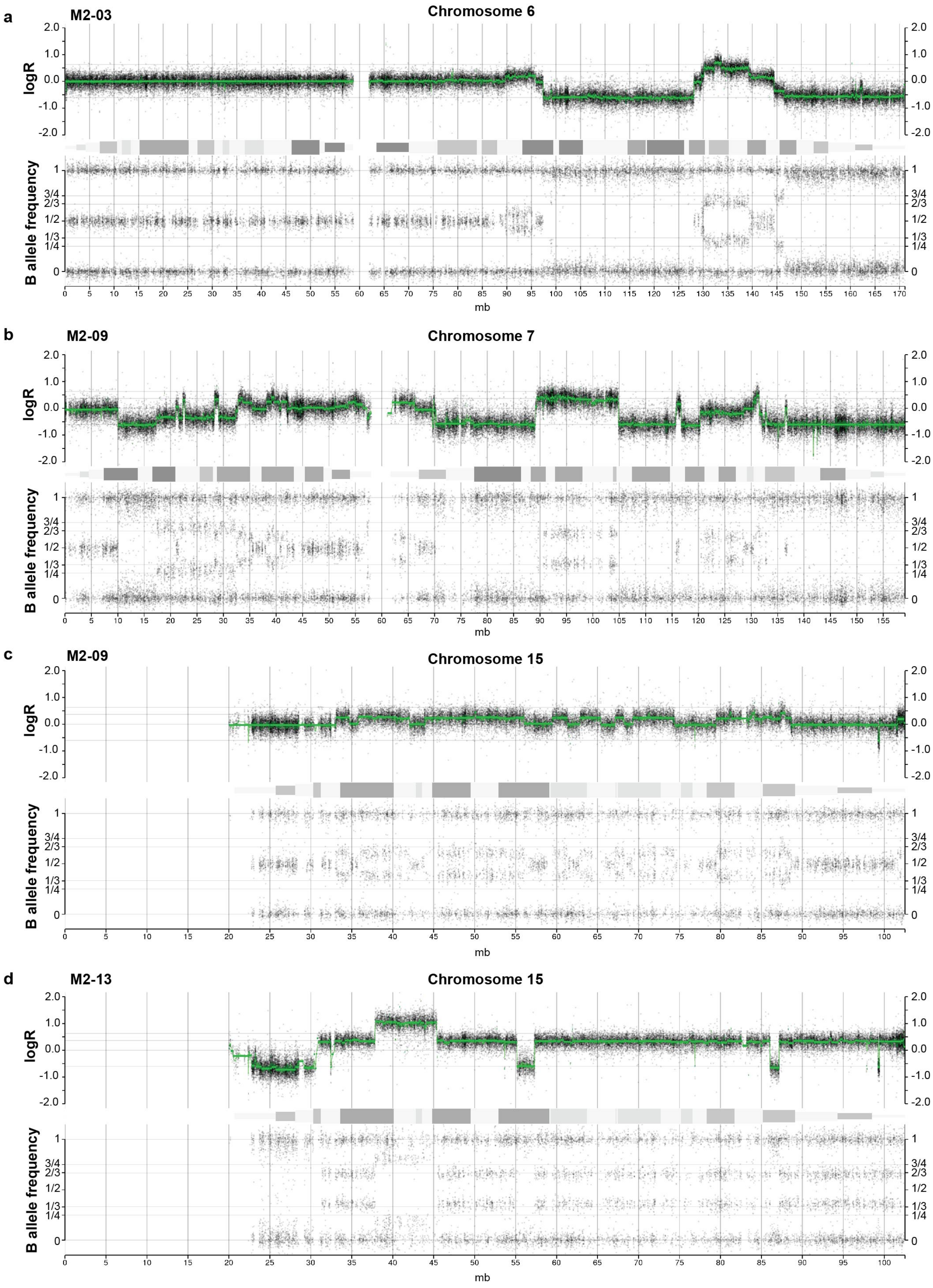

Supplementary Fig. 3. Analysis of chromothripsis in MCF10A *TP53* R273H mutant clonal cell lines.

a - d Visualization of cytogenomic microarray analysis (CMA) segmented copy number (Log_2 ratios, LogR) and B-allele frequency for the MCF10A clonal cell lines M2-03 (**a**) M2-09 (**b - c**) and M2-13 (**d**), which displayed chromothripsis or alternating segments of chromosomal gain and loss in chromosome 6 (**a**), chromosome 7 (**b**), and chromosome 15 (**c - d**).

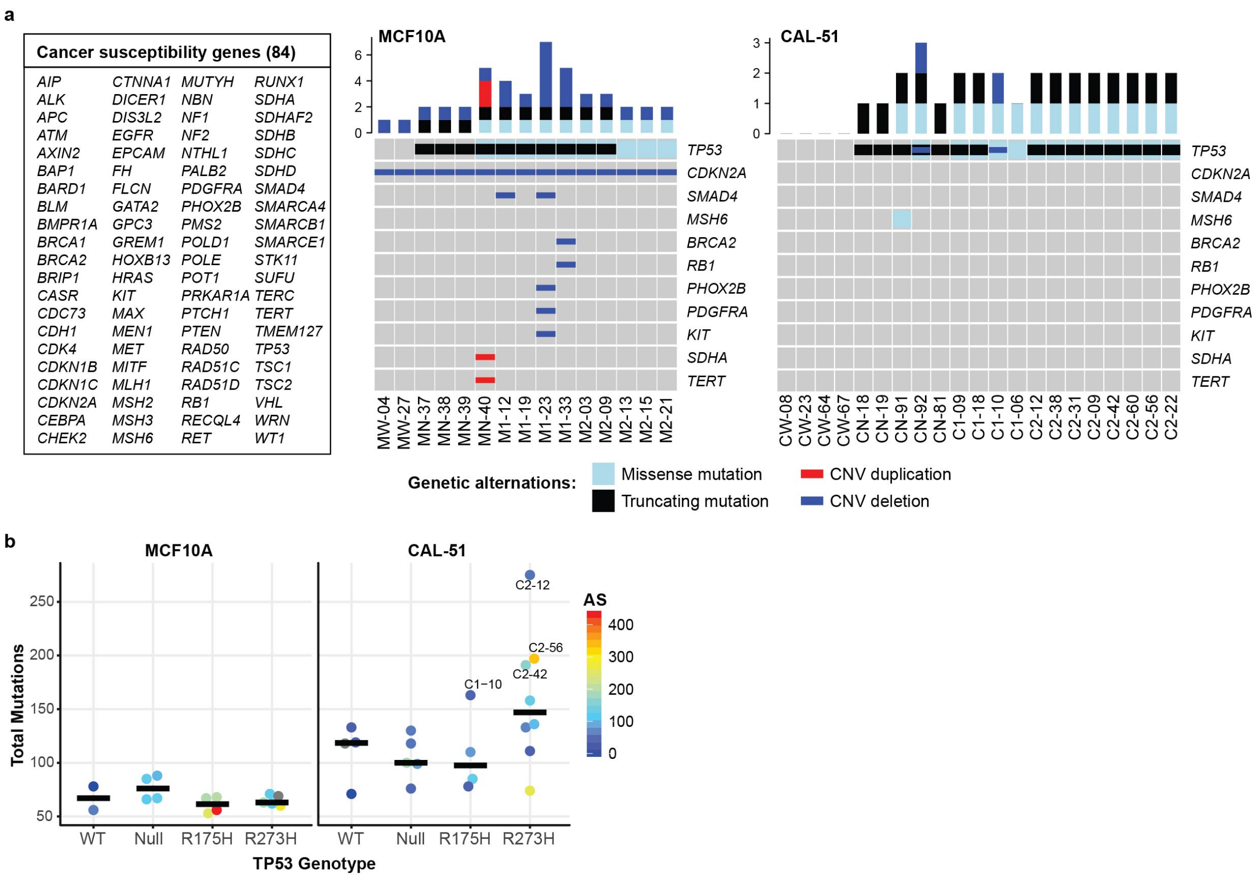

Supplementary Fig. 4. Acquisition of aneuploidy in TP53 mutant lines is not associated with other cancer driver gene mutations.

a Mutation or copy number alterations in 84 common cancer susceptibility genes (left panel) within indicated clonal cell lines from MCF10A (middle panel) and CAL-51 (right panel). Bar graphs on top of the two right panels indicate the total number of alterations within each cell line. Missense and truncating mutations are shown as light blue and black bars, respectively, in the grid below. Copy number duplication and deletion are shown as smaller red and blue bars, respectively. If a gene is not shown, no alterations were detected. **b** Dot plots comparing total number of mutations in clonal cell lines with the indicated *TP53* genotype in MCF10A (left, *n* = 2 WT, 4 Null, 4 R175H and 4 R273H) and CAL-51 (right, *n* = 4 WT, 5 Null, 4 R175H and 8 R273H) cells. Cell lines are colored by their calculated aneuploidy score (AS). Black lines indicate the median number of mutations across all clonal lines grouped according to *TP53* genotype. One-way ANOVA with Dunnett's multiple comparison test. Source data are provided in the Source Data File.

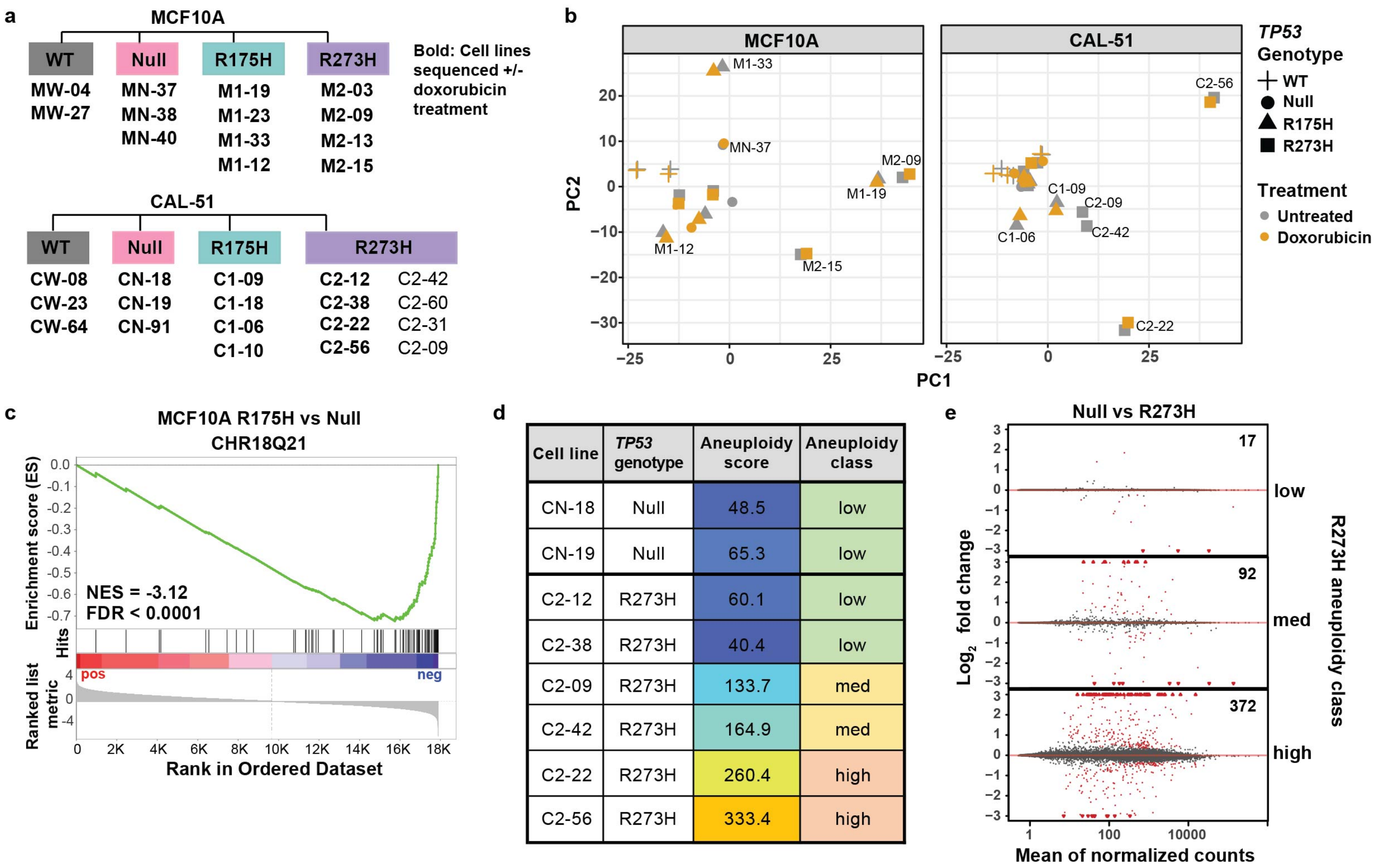

Cell line	*TP53* genotype	Aneuploidy score	Aneuploidy class
CN-18	Null	48.5	low
CN-19	Null	65.3	low
C2-12	R273H	60.1	low
C2-38	R273H	40.4	low
C2-09	R273H	133.7	med
C2-42	R273H	164.9	med
C2-22	R273H	260.4	high
C2-56	R273H	333.4	high

Supplementary Fig. 5. Gene expression changes are associated with aneuploidy and not mutant p53 expression.

a Diagram shows MCF10A (top) and CAL-51 (bottom) cell lines used for RNA-seq analyses grown under the indicated control (all cell lines) or treated (bold font; doxorubicin, 0.2 μM) conditions for 6 h. **b** PCA plot for MCF10A (left panel) and CAL-51 (right panel) clonal cell lines. Data point shapes represent the indicated *TP53* genotype (cross = WT, circle = Null, triangle = R175H, square = R273H) and treatment as described in Part A (untreated = gray, doxorubicin treated = orange). PC1, principal component 1. PC2, principal component 2. **c** GSEA plot showing negative enrichment of CHR18Q21 positional pathway genes from RNA-seq differential gene expression analysis between MCF10A *TP53* R175H and null cells. pos, positive. neg, negative. **d** Table showing CAL-51 Null and R273H cell lines separated into low, medium, or high aneuploidy classes based on their calculated aneuploidy score. **e** MA plots showing the shrunken Log_2 fold changes of all genes in CAL-51 aneuploid low, p53 null clones compared to p53 R273H mutant clones classified as having either low, medium, or high aneuploidy as

described in (**d**). The number of differentially expressed genes (*P* < 0.1, $Log_2fc > |1|$) are shown in the upper right-hand corner of each plot. Genes with *P* < 0.1 are depicted in red. *P* values are adjusted for false discovery rate. Source data are provided in the Source Data File.

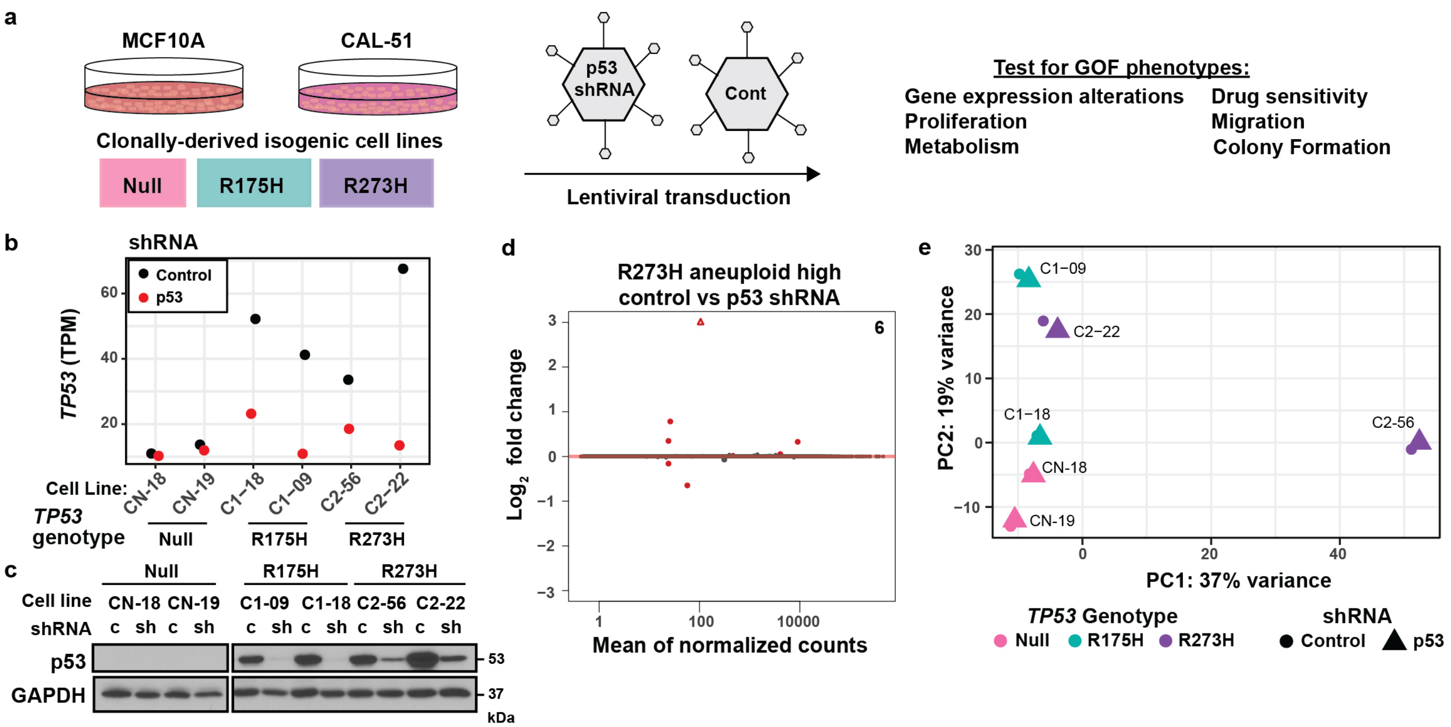

Supplementary Fig. 6. Generation of clonal lines with stable knockdown of p53 and characterization for gene expression analyses.

a Diagram demonstrating workflow of lentiviral-mediated shRNA knockdown of p53 or non-targeting control (cont) in isogenic MCF10A and CAL-51 null and *TP53* mutant (R175H and R273H) cell lines and phenotypic gain-of-function (GOF) characterization. **b** RNA-seq transcript per million (TPM) values for *TP53* in CAL-51 cells of the indicated genotype expressing p53 shRNA (p53, red) or non-targeting shRNA controls (control, black). **c** Western blot analysis of p53 and GAPDH protein in CAL-51 cells with p53 shRNA (sh) or non-targeting control shRNA (c). Blots are representative of at least two independent experiments. **d** MA plot showing the shrunken Log_2 fold change of all genes in CAL-51 R273H aneuploid high cells (C2-56 and C2-22) containing stable expression of p53 shRNA compared to the same cells containing non-targeting shRNA controls. The number of differentially expressed genes (False Discovery Rate adjusted $P < 0.1$, $Log_2fc > |1|$) are shown in the upper right-hand corner of each plot. Genes with $P < 0.1$ are depicted in red. **e** PCA plot for CAL-51 cells containing p53 shRNA (triangle) or non-targeting shRNA controls (control, circle). Source data are provided in the Source Data File.

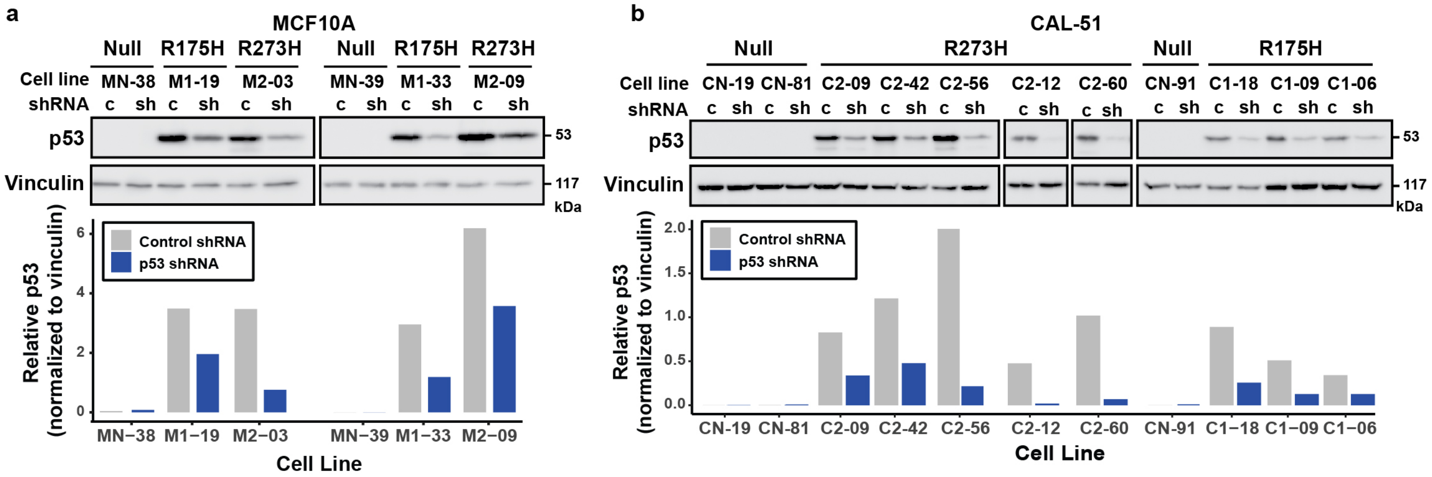

Supplementary Fig. 7. Analysis of p53 protein in MCF10A and CAL-51 clonal lines with stable p53 knockdown.

a - b Western blot analyses of the indicated proteins in the indicated MCF10A (**a**) and CAL-51 (**b**) cells with p53 shRNA (sh) or non-targeting control shRNA (c). Lower panels display quantification of p53 protein levels (normalized to the loading control vinculin) and correspond to upper panels (**a – b**). Blots are representative of at least two independent experiments. Source data are provided in the Source Data File.

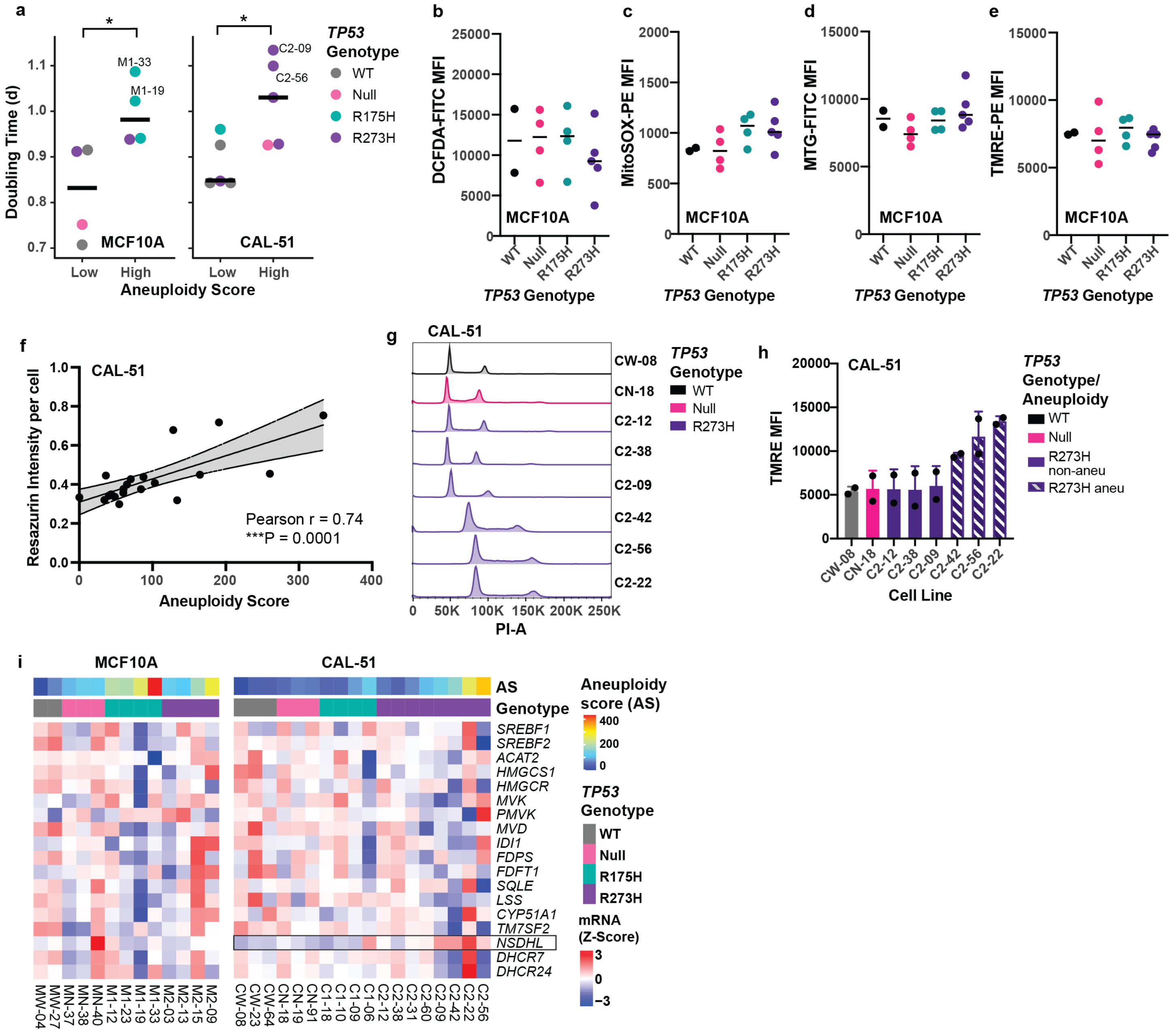

Supplementary Fig. 8. Analysis of proliferation and metabolic activity in isogenic clonally-derived lines.

a Doubling time in MCF10A (left) and CAL-51 (right) cells classified by aneuploidy score (AS) as either aneuploid-low (lower quartile AS) or aneuploid-high (upper quartile AS). Dots represent the mean for each cell line from at least two independent experiments. Bar indicates median value per group. **P* = 0.0348, 0.0469, from left to right, Two-tailed student's t-test. **b - e** Dot plots showing intensity of the metabolic indicators (**b**) DCFDA (reactive oxygen species) (**c**) MitoSOX (mitochondrial

superoxides) (**d**) MTG (mitochondrial mass) and (**e**) TMRE (mitochondrial membrane potential), across all MCF10A cell lines with the indicated *TP53* genotype (n = 2 WT, 4 Null, 4 R175H, and 5 R273H). Each point represents the average geometric mean fluorescent intensity (MFI). Bars indicate the median staining for each genotype from two independent experiments. **f** Scatter plot showing aneuploidy score versus the average resazurin staining intensity for all CAL-51 cell lines. The black line represents a linear model of the best fit, with the gray area representing the 95% confidence intervals. Significance was determined using Pearson correlation, ***P = 0.0001. **g** Cell cycle distributions showing DNA content of cells in (**h**) by propidium iodide (PI) staining and flow cytometry in the CAL-51 isogenic model. Colors represent *TP53* genotype (black = WT, pink = Null, purple = R273H). **h** Bar graph showing TMRE mean fluorescent intensity of CAL-51 non-aneuploid (solid purple) and aneuploid (dashed purple) R273H cell lines. Mean + s.d. from n = 2 independent experiments. **i** Heatmaps comparing gene expression for mevalonate pathway transcription factors and genes encoding for pathway enzymes in MCF10A (left) and CAL-51 (right) cell lines. Source data are provided in the Source Data File.

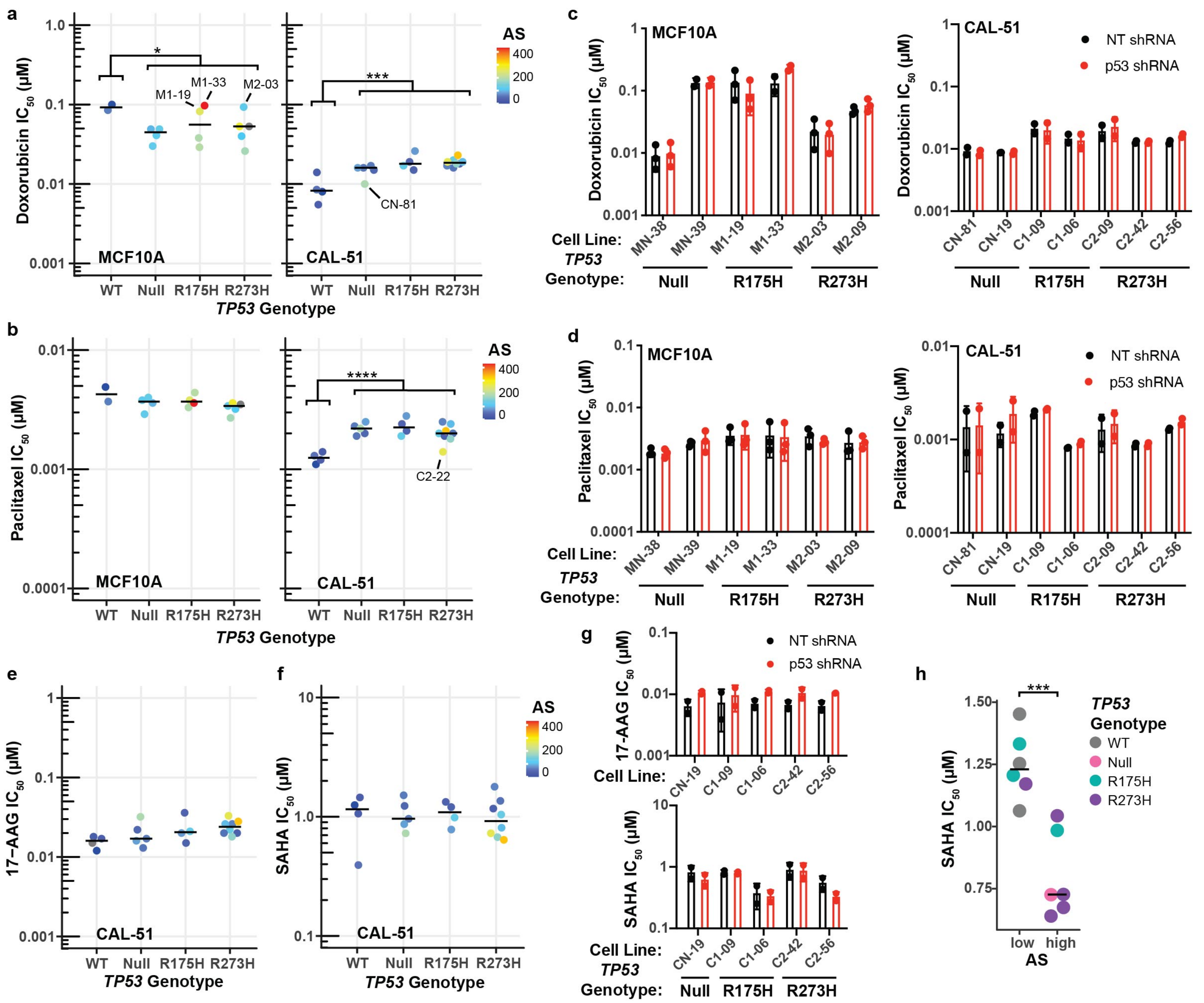

Supplementary Fig. 9: Clonal differences in drug sensitivity are associated with aneuploidy and not mutant p53 expression.

a, b IC_{50} values for MCF10A (left) and CAL-51 (right) cell lines treated for 72 h with increasing concentrations of either doxorubicin (**a**) or paclitaxel (**b**). **c, d** IC_{50} values in MCF10A (left) and CAL-51 (right) cell lines containing non-targeting control (NT, black) or p53 shRNAs (red) after treatment with doxorubicin (**c**) or paclitaxel (**d**). Mean ± s.d. from n = 2 two independent experiments per CAL-51 cell line, or n = 3 for MCF10A cell lines (except for MN-39, and M1-33 in (**c**), n = 2). **e, f** IC_{50} values for CAL-51 cell lines treated for 72 h with increasing concentrations of either 17-AAG (**e**) or SAHA (**f**). **g** Mean 17-AAG (top) and SAHA (bottom) IC_{50} values in CAL-51 cell lines containing non-targeting (NT, black) or p53 shRNAs (red). Mean ± s.d. from n = 2 independent experiments per cell

line. **h** Mean SAHA IC_{50} values for *n* = 6 high-aneuploid and *n* = 6 low-aneuploid (upper and lower quartile AS, respectively) CAL-51 cell lines. Colors indicate *TP53* genotype. Two-tailed student's t-test, ****P* = 0.0005. (**a, b, e, f**) Dots represent the mean for each cell line (MCF10A *n* = 2 WT, 4 Null, 4 R175H, and 5 R273H cell lines; CAL-51 *n* = 4 WT, 5 Null, 4 R175H and 8 R273H cell lines) from at least two independent experiments. Bars represent the median per *TP53* genotype. Dots are colored by their calculated aneuploidy score (AS), and those colored in gray were not profiled in cytogenomic microarray experiments. **P* < 0.05, ****P* < 0.001, *****P* < 0.0001, Two-tailed student's t-tests. Significance between *TP53* genotypes tested using one-way ANOVA with Dunnett's multiple comparison test. (**c, d, g**) Significance tested using two-way analysis of variance with Sidak's multiple comparisons test. Western blots showing knockdown of p53 are shown in Supplementary Fig. 7b, c. Source data, values of *n* and exact *P* values are provided in the Source Data File.

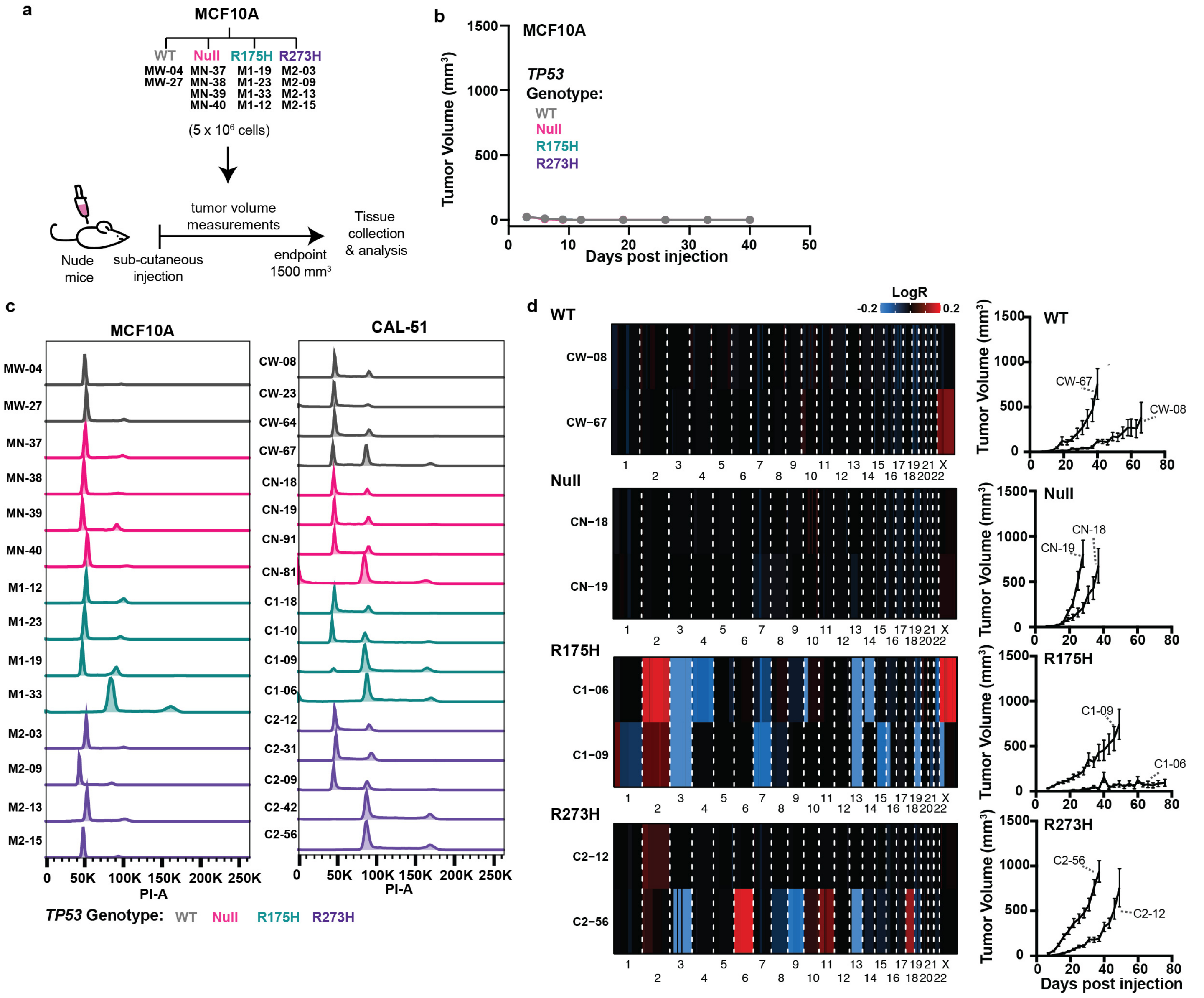

Supplementary Fig. 10. Analysis of tumorigenicity and aneuploidy in MCF10A and CAL-51 cells.

a Diagram demonstrating workflow for MCF10A xenograft tumor growth experiment. **b** Tumor volume (in cubic millimeters) of MCF10A cell lines. Data shown represent the mean ± s.e.m. of tumor volume (n = 10 tumors per cell line) measured every three days (for palpable tumors) or weekly for mice with no detectable tumor across MCF10A cells indicated in (**a**) by *TP53* genotype. **c** Histograms showing DNA content of injected MCF10A (left) and CAL-51 (right) xenograft cells by flow cytometry of propidium iodide (PI) stained cells. Colors represent *TP53* genotype. **d** Copy number alterations from cytogenomic microarray analyses of tumor DNA (left) from select

corresponding CAL-51 cell line xenografts (right). Xenograft growth was measured as tumor volume (in cubic millimeters) and represents the mean ± s.e.m of *n* = 10 tumors per cell line. Chromosomal gain (red) and loss (blue). Source data are provided in the Source Data File.

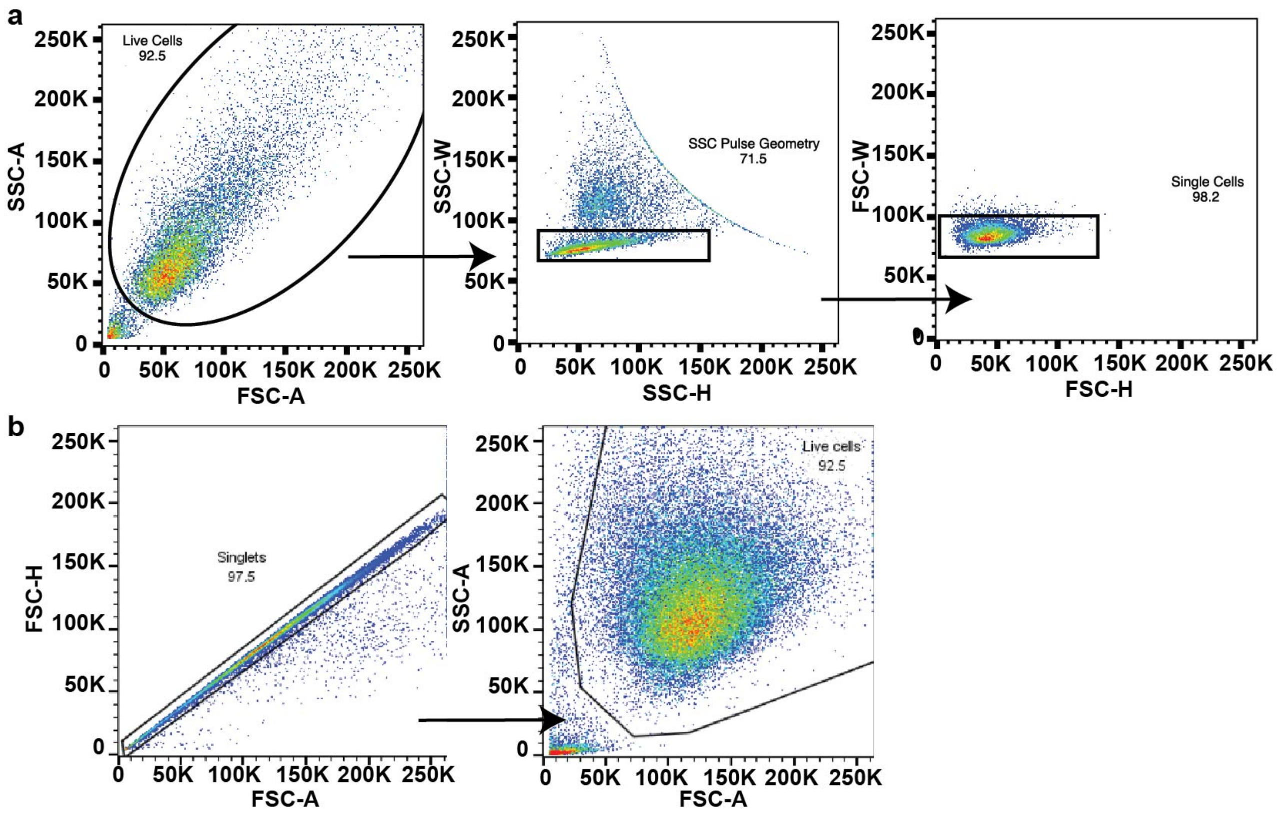

Supplementary Fig. 11. Gating strategies for DNA content and metabolic staining analyses.

a Gating strategy for DNA content analyses of MCF10A and CAL-51 isogenic clonal cell lines shown in Supplemental Fig. 2c, 8g, and 10c. **b** Gating strategy for metabolic staining experiments shown in Supplementary Fig. 8b-e.

Supplementary Table 1. Validation of *TP53* alterations in isogenic cell lines by whole-exome-sequencing.

		Allele 1				Allele 2 (for heterozygous alterations)			
Model	Cell Line	Variant Classification	Alteration	Consequence	AF	Variant Classification	Alteration	Consequence	AF
MCF10A	MW-04[a]								
MCF10A	MW-27	synonymous SNV<br>synonymous SNV	c.C828T:p.A276A<br>c.T825C:p.C275C	SILENT<br>SILENT					
MCF10A	MN-37	frameshift deletion	c.830_831del:p.C277fs	TRUNCATING	0.45	frameshift insertion	c.831dupT:p.P278fs	TRUNCATING	0.55
MCF10A	MN-38	frameshift deletion	c.830_831del:p.C277fs	TRUNCATING	0.54	frameshift insertion	c.831dupT:p.P278fs	TRUNCATING	0.46
MCF10A	MN-39	frameshift deletion	c.831_837del:p.C277fs	TRUNCATING	0.99				
MCF10A	MN-40	synonymous SNV<br>synonymous SNV<br>nonsynonymous SNV<br>frameshift insertion	c.C828T:p.A276A<br>c.T825C:p.C275C<br>c.C817A:p.R273S<br>c.818_819insGTGCA:p.R273fs	SILENT<br>SILENT<br>MISSENSE<br>TRUNCATING	 <br>0.36<br>0.35	nonsynonymous SNV<br>frameshift deletion	c.T831G:p.C277W<br>c.832_833del:p.P278fs	MISSENSE<br>TRUNCATING	0.59<br>0.59
MCF10A	M1-12	synonymous SNV<br>nonsynonymous SNV	c.C531A:p.P177P<br>c.G524A:p.R175H	SILENT<br>MISSENSE	 0.47	stop gain	c.537dupT:p.E180_R181delinsX	TRUNCATING	0.53
MCF10A	M1-19	synonymous SNV<br>nonsynonymous SNV	c.C531A:p.P177P<br>c.G524A:p.R175H	SILENT<br>MISSENSE	 0.54	frameshift insertion	c.536_537insC:p.H179fs	TRUNCATING	0.46
MCF10A	M1-23	synonymous SNV<br>nonsynonymous SNV	c.C531A:p.P177P<br>c.G524A:p.R175H	SILENT<br>MISSENSE	 0.49	frameshift insertion	c.536dupA:p.H179fs	TRUNCATING	0.52
MCF10A	M1-33	synonymous SNV<br>nonsynonymous SNV	c.C531A:p.P177P<br>c.G524A:p.R175H	SILENT<br>MISSENSE	 0.49	nonsynonymous SNV<br>frameshift deletion	c.T537A:p.H179Q<br>c.538delG:p.E180fs	MISSENSE<br>TRUNCATING	0.51<br>0.51
MCF10A	M2-03	synonymous SNV<br>synonymous SNV<br>nonsynonymous SNV	c.C828T:p.A276A<br>c.T825C:p.C275C<br>c.G818A:p.R273H	SILENT<br>SILENT<br>MISSENSE	 <br>0.48	frameshift deletion	c.818delG:p.R273fs	TRUNCATING	0.52
MCF10A	M2-09	synonymous SNV<br>synonymous SNV<br>nonsynonymous SNV	c.C828T:p.A276A<br>c.T825C:p.C275C<br>c.G818A:p.R273H	SILENT<br>SILENT<br>MISSENSE	 <br>0.41	frameshift deletion	c.830_831del:p.C277fs	TRUNCATING	0.57
MCF10A	M2-13	synonymous SNV<br>synonymous SNV<br>nonsynonymous SNV	c.C828T:p.A276A<br>c.T825C:p.C275C<br>c.G818A:p.R273H	SILENT<br>SILENT<br>MISSENSE	 <br>1				
MCF10A	M2-15	synonymous SNV<br>synonymous SNV<br>nonsynonymous SNV	c.C828T:p.A276A<br>c.T825C:p.C275C<br>c.G818A:p.R273H	SILENT<br>SILENT<br>MISSENSE	 <br>1				
MCF10A	M2-21	synonymous SNV<br>synonymous SNV<br>nonsynonymous SNV	c.C828T:p.A276A<br>c.T825C:p.C275C<br>c.G818A:p.R273H	SILENT<br>SILENT<br>MISSENSE	 <br>1				
CAL-51	CW-08[a]								
CAL-51	CW-23	synonymous SNV<br>synonymous SNV	c.C828T:p.A276A<br>c.T825C:p.C275C	SILENT<br>SILENT					
CAL-51	CW-64	synonymous SNV<br>synonymous SNV	c.C828T:p.A276A<br>c.T825C:p.C275C	SILENT<br>SILENT					
CAL-51	CW-67	synonymous SNV<br>synonymous SNV	c.C828T:p.A276A<br>c.T825C:p.C275C	SILENT<br>SILENT					
CAL-51	CN-18	frameshift deletion	c.586delC:p.R196fs	TRUNCATING	0.98				
CAL-51	CN-19	frameshift insertion	c.586dupC:p.R196fs	TRUNCATING	1				
CAL-51	CN-91	frameshift deletion	c.586delC:p.R196fs	TRUNCATING	1				
CAL-51	CN-92	frameshift deletion	c.583_586del:p.I195fs	TRUNCATING	1				
CAL-51	CN-81	frameshift deletion	c.536_546del:p.H179fs	TRUNCATING	1				
CAL-51	C1-09	synonymous SNV<br>nonsynonymous SNV	c.C531A:p.P177P<br>c.G524A:p.R175H	SILENT<br>MISSENSE	 0.56	frameshift insertion	c.536_537insC:p.H179fs	TRUNCATING	0.44
CAL-51	C1-18	synonymous SNV<br>nonsynonymous SNV	c.C531A:p.P177P<br>c.G524A:p.R175H	SILENT<br>MISSENSE	 0.58	frameshift insertion	c.536_537insC:p.H179fs	TRUNCATING	0.39
CAL-51	C1-10	synonymous SNV<br>nonsynonymous SNV	c.C531A:p.P177P<br>c.G524A:p.R175H	SILENT<br>MISSENSE	 1				
CAL-51	C1-06	synonymous SNV<br>nonsynonymous SNV	c.C531A:p.P177P<br>c.G524A:p.R175H	SILENT<br>MISSENSE	 0.87				
CAL-51	C2-09	synonymous SNV<br>synonymous SNV<br>nonsynonymous SNV	c.C828T:p.A276A<br>c.T825C:p.C275C<br>c.G818A:p.R273H	SILENT<br>SILENT<br>MISSENSE	 <br>0.35	frameshift deletion<br>frameshift deletion	c.830_831del:p.C277fs<br>c.826_827del:p.A276fs	TRUNCATING<br>TRUNCATING	0.63<br>0.64
CAL-51	C2-12	synonymous SNV<br>synonymous SNV<br>nonsynonymous SNV	c.C828T:p.A276A<br>c.T825C:p.C275C<br>c.G818A:p.R273H	SILENT<br>SILENT<br>MISSENSE	 <br>0.45	frameshift deletion	c.830_831del:p.C277fs	TRUNCATING	0.52
CAL-51	C2-31	synonymous SNV<br>synonymous SNV<br>nonsynonymous SNV	c.C828T:p.A276A<br>c.T825C:p.C275C<br>c.G818A:p.R273H	SILENT<br>SILENT<br>MISSENSE	 <br>0.48	frameshift insertion	c.830dupG:p.C277fs	TRUNCATING	0.53
CAL-51	C2-38	synonymous SNV<br>synonymous SNV<br>nonsynonymous SNV	c.C828T:p.A276A<br>c.T825C:p.C275C<br>c.G818A:p.R273H	SILENT<br>SILENT<br>MISSENSE	 <br>0.53	frameshift deletion	c.831delT:p.C277fs	TRUNCATING	0.48
CAL-51	C2-42	synonymous SNV<br>synonymous SNV<br>nonsynonymous SNV	c.C828T:p.A276A<br>c.T825C:p.C275C<br>c.G818A:p.R273H	SILENT<br>SILENT<br>MISSENSE	 <br>0.49	stop gain	c.830_831insA:p.C277_P278delinsX	TRUNCATING	0.51
CAL-51	C2-56	synonymous SNV<br>synonymous SNV<br>nonsynonymous SNV	c.C828T:p.A276A<br>c.T825C:p.C275C<br>c.G818A:p.R273H	SILENT<br>SILENT<br>MISSENSE	 <br>0.50	frameshift deletion	c.830delG:p.C277fs	TRUNCATING	0.50
CAL-51	C2-60	synonymous SNV<br>synonymous SNV<br>nonsynonymous SNV	c.C828T:p.A276A<br>c.T825C:p.C275C<br>c.G818A:p.R273H	SILENT<br>SILENT<br>MISSENSE	 <br>0.51	frameshift deletion	c.831delT:p.C277fs	TRUNCATING	0.47
CAL-51	C2-22	synonymous SNV<br>synonymous SNV<br>nonsynonymous SNV	c.C828T:p.A276A<br>c.T825C:p.C275C<br>c.G818A:p.R273H	SILENT<br>SILENT<br>MISSENSE	 <br>0.50	frameshift deletion	c.818delG:p.R273fs	TRUNCATING	0.50

Annotation based on NM_000546. AF = allele frequency. Allele frequencies for synonymous mutations are indicated. [a]Parental WT clonal cell line for each model.

Supplementary Table 2. Analysis of mutations common to aneuploid high isogenic cell lines.

Symbol	Aneuploid High (n)	Aneuploid Low (n)	P-value	Adj P-value	Model	Cell Lines Altered
TP53	4	2	0.42857	1	MCF10A	MN-37, MN-38, MN-39, MN-40, M1-12, M1-19, M1-23, M1-33, M2-03, M2-09, M2-13, M2-15, M2-21
HKDC1	4	2	0.42857	1	MCF10A	MW-04, MN-37, MN-38, M1-12, M1-19, M1-23, M1-33, M2-09, M2-21
HTT	2	4	0.42857	1	MCF10A	MW-04, MW-27, MN-37, MN-39, MN-40, M1-19, M2-03, M2-09, M2-13, M2-21
NCSTN	0	2	0.42857	1	MCF10A	MW-04, MN-37, MN-38, M2-21
RYR2	0	2	0.42857	1	MCF10A	MW-04, MN-37, MN-38, M2-21
WNK3	0	2	0.42857	1	MCF10A	MW-04, MN-37, MN-38, M2-21
SYN2	3	1	0.48571	1	MCF10A	MN-37, MN-40, M1-12, M1-19, M1-23, M1-33, M2-21
TP53	5	2	0.16667	1	CAL-51	CN-18, CN-19, CN-91, CN-92, CN-81, C1-09, C1-18, C1-10, C1-06, C2-09, C2-12, C2-31, C2-38, C2-42, C2-56, C2-60, C2-22
PXDN	0	3	0.16667	1	CAL-51	CW-08, CW-64, CN-19, C1-18
BICRA	1	3	0.52381	1	CAL-51	CW-08 CW-64, CN-18, CN-19, C1-18, C2-12, C2-22
KCNN3	3	1	0.52381	1	CAL-51	CN-92, C1-06,C2-38, C2-42, C2-56, C2-22
TTN	3	1	0.52381	1	CAL-51	CN-18, CN-81, C2-12, C2-38, C2-42, C2-56, C2-22
VCX3B	4	2	0.52381	1	CAL-51	CW-08, CW-23, CW-67, CN-18 CN-19, C1-10, C1-06, C2-09, C2-12, C2-31, C2-42, C2-56, C2-22

Analysis of whole-exome sequencing and two-sided Fischer's Exact Test between mutations in aneuploid-high (upper quartile AS) and aneuploid-low (lower quartile AS) cell lines in MCF10A and CAL-51 isogenic cell line models.

Supplementary Table 3. TCGA cohort acronyms and the number of tumors analyzed.

Study Abbreviation	Study Name	Tumors (total)	Tumors per indicated TP53 Genotype		
			WT	Truncating	Missense
BLCA	Bladder urothelial carcinoma	344	196	51	97
BRCA	Breast invasive carcinoma	985	658	130	197
COAD	Colon adenocarcinoma	339	163	40	136
ESCA	Esophageal carcinoma	131	17	44	70
GBM	Glioblastoma multiforme	333	244	16	73
HNSC	Head and Neck squamous cell carcinoma	352	128	102	122
LGG	Brain lower grade glioma	425	255	23	147
LIHC	Kidney renal papillary cell carcinoma	342	245	37	60
LUAD	Lung adenocarcinoma	432	220	75	137
LUSC	Lung squamous cell carcinoma	380	55	118	207
OV	Ovarian serous cystadenocarcinoma	352	29	125	198
PAAD	Pancreatic adenocarcinoma	152	55	39	58
PRAD	Prostate adenocarcinoma	461	409	16	36
READ	Rectum adenocarcinoma	128	36	30	62
SARC	Sarcoma	231	153	37	41
SKCM	Skin cutaneous Melanoma	409	345	28	36
STAD	Stomach adenocarcinoma	376	201	69	106
UCEC	Uterine corpus endometrial carcinoma	461	304	40	117
UCS	Uterine carcinosarcoma	49	5	8	36

Supplementary Table 4. Primers utilized in this study.

Name	Sequence
R175H & Null FWD	CACTTGTGCCCTGACTTTCA
R175H & Null REV	TTGCACATCTCATGGGGTTA
R273H FWD	CCTCTGCTTGCCTCTGACCCCT
R273H REV	TGCACCCTTGGTCTCCTCCACC
