# Supplementary material for: Acquisition of aneuploidy drives mutant p53-associated gain-of-function phenotypes
Source: Nat Commun. 2021 Aug 31;12:5184. doi: 10.1038/s41467-021-25359-z (PMC8408227; doi:10.1038/s41467-021-25359-z)
Supplement: Supplementary file 2 — Description of Additional Supplementary Files [file 41467_2021_25359_MOESM2_ESM.pdf]

Description of Additional Supplementary Files

File Name: Supplementary Data 1
Description: Differential gene expression between MCF10A and CAL-51 mutant and null isogenic cell lines. Differentially expressed genes (False discovery rate adjusted $P < 0.1$, absolute log2fc >= 1) in MCF10A and CAL-51 R175H and R273H mutant compared to Null cells. Experiments were conducted at passage 30 (with and without doxorubicin treatment).
