# Supplementary material for: Acquisition of aneuploidy drives mutant p53-associated gain-of-function phenotypes
Source: Nat Commun. 2021 Aug 31;12:5184. doi: 10.1038/s41467-021-25359-z (PMC8408227; doi:10.1038/s41467-021-25359-z)
Supplement: Supplementary file 4 — Reporting Summary [file 41467_2021_25359_MOESM4_ESM.pdf]

nature research

Corresponding author(s): Jennifer A. Pietenpol

Last updated by author(s): Jul 19, 2021

Reporting Summary

Nature Research wishes to improve the reproducibility of the work that we publish. This form provides structure for consistency and transparency in reporting. For further information on Nature Research policies, see our Editorial Policies and the Editorial Policy Checklist.

Statistics

For all statistical analyses, confirm that the following items are present in the figure legend, table legend, main text, or Methods section.

n/a	Confirmed	
☐	☒	The exact sample size (*n*) for each experimental group/condition, given as a discrete number and unit of measurement
☐	☒	A statement on whether measurements were taken from distinct samples or whether the same sample was measured repeatedly
☐	☒	The statistical test(s) used AND whether they are one- or two-sided<br>*Only common tests should be described solely by name; describe more complex techniques in the Methods section.*
☒	☐	A description of all covariates tested
☐	☒	A description of any assumptions or corrections, such as tests of normality and adjustment for multiple comparisons
☐	☒	A full description of the statistical parameters including central tendency (e.g. means) or other basic estimates (e.g. regression coefficient) AND variation (e.g. standard deviation) or associated estimates of uncertainty (e.g. confidence intervals)
☐	☒	For null hypothesis testing, the test statistic (e.g. *F*, *t*, *r*) with confidence intervals, effect sizes, degrees of freedom and *P* value noted<br>*Give P values as exact values whenever suitable.*
☒	☐	For Bayesian analysis, information on the choice of priors and Markov chain Monte Carlo settings
☒	☐	For hierarchical and complex designs, identification of the appropriate level for tests and full reporting of outcomes
☐	☒	Estimates of effect sizes (e.g. Cohen's *d*, Pearson's *r*), indicating how they were calculated

Our web collection on statistics for biologists contains articles on many of the points above.

Software and code

Policy information about availability of computer code

Data collection	MetaXpress Multi-Wavelength Cell Scoring Module V6.6.3.733 (Molecular Devices)<br>Gen5 (Biotek)
Data analysis	For RNA-seq analysis: Flexbar v3.4.0 was used to remove adapter sequences; FastQC v0.11.7 and MultiQC v1.5 were used to evaluate quality; Salmon v0.12.0 was used to quantify transcript abundance; tximport 1.12.3, DESeq2 v1.24.0, IHW_1.12.0, and ashr_2.2-47 in R v3.6.1 were used for differential gene expression analysis; and GSEA v4.0.3 was used for gene set enrichment analysis. FlowJo (10.0.7 and 10.5.3) was used for gating and visualization of flow cytometry of propidium iodide staining and metabolic staining experiments. Fiji (2.1.0) was used for quantification of western blots and manual counting of metaphase spreads. For copy number analysis: rawcopy_1.1 R package (http://rawcopy.org) was used to process raw .CEL files; copynumber v_1.24.0 R package was used for visualization. CNApp was used for statistical analysis (https://tools.idibaps.org/CNApp/). For whole exome sequencing an NGSPERL based custom pipeline was used: Cutadapt (2.10) was used to trim reads; BWA (0.7.17-r1188) was used for alignment; GATK3 and GATK4 was used for refinment and calling SNVs, respectively; ANNOVAR (20180416) was used for annotating SNVs; and the R maftools package was used for mutation analysis. Prism (Graphpad, 8.4.3) was used for IC50 calculations, visualization, and statistical analysis. CellProfiler (3.1.9) was used for quantification of transwell migration assays. Integrative Genomics Viewer (IGV 2.3.97) was used for visualization of MCF10A and CAL-51 copy number profiles. R 4.0.0 was used for analysis of TCGA data, survival analysis with the R survival package, and visualization.<br><br>The code used in this study is available from the corresponding author upon reasonable request and at https://github.com/shengqh/TP53_isogenics_exomeseq_3772_hg38.

For manuscripts utilizing custom algorithms or software that are central to the research but not yet described in published literature, software must be made available to editors and reviewers. We strongly encourage code deposition in a community repository (e.g. GitHub). See the Nature Research guidelines for submitting code & software for further information.

Data

Policy information about availability of data

All manuscripts must include a data availability statement. This statement should provide the following information, where applicable:

- Accession codes, unique identifiers, or web links for publicly available datasets
- A list of figures that have associated raw data
- A description of any restrictions on data availability

The cytogenomic and processed sequencing data that support the findings of this study are available at https://data.mendeley.com/datasets/vr8fcbczz5/2 [DOI: 10.17632/vr8fcbczz5.2]. The raw sequencing data that support the findings of this study have been deposited to the Sequence Read Archive under the BioProject accession number PRJNA669391 [https://www.ncbi.nlm.nih.gov/bioproject/PRJNA669391]. All other data supporting the findings of this study are available within the article, its supplementary information files and the source data provided with this paper. Publicly available data used in this manuscript were obtained from Public MC3 TCGA MAF, https://gdc.cancer.gov/about-data/publications/mc3-2017; TCGA Survival Data, https://gdc.cancer.gov/about-data/publications/pancanatlas; CCLE, https://portals.broadinstitute.org/ccle and https://depmap.org/portal/download/; MCF10A copy number data, https://www.synapse.org/ - ! Synapse:syn2346643/wiki/62255 and MetMap data, https://depmap.org/metmap/data/index.html.

Field-specific reporting

Please select the one below that is the best fit for your research. If you are not sure, read the appropriate sections before making your selection.

☒ Life sciences ☐ Behavioural & social sciences ☐ Ecological, evolutionary & environmental sciences

For a reference copy of the document with all sections, see nature.com/documents/nr-reporting-summary-flat.pdf

Life sciences study design

All studies must disclose on these points even when the disclosure is negative.

Sample size	No statistical method was used to determine sample size for in vitro experiments. Sample size for in vivo experiments was calculated using a power analysis. Analyses included as many independent clonal cell lines (biological replicates) as experimentally feasible.
Data exclusions	No data were excluded from the analyses.
Replication	Experiments were independently repeated a minimum of two times and reliably reproduced across multiple clonal cell lines as indicated in the figure legends.
Randomization	Randomization was not relevant for in-vitro experiments because findings were based on predetermined grouping of all available cell lines by TP53 genotype or by knockdown conditions (non-targeting or TP53 shRNA). The order in which cell lines were seeded for experiments was done at random. For in vivo tumor xenograft experiments, animals were randomly assigned to cages and experimental conditions. For cell line xenograft injections, the order in which injections were performed was random.
Blinding	Blinding of cell line genotype and aneuploidy status was performed for in vitro metabolic staining experiments, quantification of transwell migration assays and colony formation assays, whole exome sequencing data analysis, and when possible, for quantification of metaphase spreads. For in vivo tumor xenograft experiments, injections and tumor volume measurements were performed with cell line genotype and aneuploidy status blinded to the experimenter. For the in vitro experiments not listed here, investigators were not blinded because the results of measurements or sequencing experiments were not affected by knowledge of cell line identities or aneuploidy status.

Reporting for specific materials, systems and methods

We require information from authors about some types of materials, experimental systems and methods used in many studies. Here, indicate whether each material, system or method listed is relevant to your study. If you are not sure if a list item applies to your research, read the appropriate section before selecting a response.

Materials & experimental systems

n/a	Involved in the study	
☐	☒	Antibodies
☐	☒	Eukaryotic cell lines
☒	☐	Palaeontology and archaeology
☐	☒	Animals and other organisms
☒	☐	Human research participants
☒	☐	Clinical data
☒	☐	Dual use research of concern

Methods

n/a	Involved in the study	
☒	☐	ChIP-seq
☐	☒	Flow cytometry
☒	☐	MRI-based neuroimaging

Antibodies

Antibodies used	Mouse monoclonal anti-p53 (DO-1), Santa Cruz, Cat# sc-126, RRID:AB_628082; Mouse monoclonal anti-p21 (F-5), Santa Cruz, Cat# sc-6246, RRID:AB_628073; Mouse monoclonal anti-MDM2 (SMP14), Santa Cruz, Cat# sc-965, RRID:AB_627920; Mouse monoclonal anti-GAPDH, Merck Millipore, MAB374, 1:10000; Mouse monoclonal anti-β-Actin (C4), Santa Cruz, Cat# sc-47778, RRID:AB_626632; Rabbit monoclonal anti-Vinculin (42H89L44), Invitrogen, Cat# 700062, RRID:AB_2532280; Goat anti-Mouse IgG, HRP-conjugated secondary, Thermo Fischer Scientific, Cat# 31432; RRID:AB_228302; Goat anti-Rabbit IgG, HRP-conjugated secondary, Thermo Fischer Scientific, Cat# 31462, RRID:AB_228338.
Validation	Anti-p53 Antibody (DO-1) is recommended for detection of wild type and mutant p53 under denaturing and non-denaturing conditions of mouse, rat and human origin by WB, IP, IF, IHC(P) and FCM. Specificity of this antibody is also validated through use of our WT, null or mutant p53 cell lines.<br>Anti-p21 Antibody (F-5) is recommended for detection of p21 of mouse, rat and human origin by WB, IP, IF, IHC(P) and FCM. Specificity of this antibody is also validated through use of our WT, null or mutant p53 cell lines.<br>Anti-MDM2 Antibody (SMP14) is recommended for detection of MDM2, MDM2 p60 cleavage product and p53-MDM2 complexes of mouse, rat and human origin by WB, IP, IF and IHC(P). Specificity of this antibody is also validated through use of our WT, null or mutant p53 cell lines.<br>Anti-Glyceraldehyde-3-Phosphate Dehydrogenase (GAPDH) Antibody, clone 6C5 is a well published and extensively characterized monoclonal antibody. This purified mAb detects Glyceraldehyde-3-Phosphate Dehydrogenase (GAPDH) & has been published & validated for use in ELISA, IP, IC, IF, IH & WB. It recognizes a 36kDa band of the reduced monomer. Non-reduced GAPDH runs as a 146kDa tetramer. Specificity: Glyceraldehyde-3-phosphate dehydrogenase (GAPDH) from skeletal muscle. Antibody also recognizes cardiac GAPDH. GAPDH enzyme is detected in many non-muscle cells lines including HeLa, HCT-116 cells, U937 and THP-1 cells among others. For quality assurance this antibody was evaluated by Western Blot on A431 lysates.<br>Anti-beta-Actin Antibody (C4) is recommended for detection of β-Actin of mouse, rat, human, avian, bovine, canine, porcine, rabbit, Dictyostelium discoideum and Physarum polycephalum origin by WB, IP, IF, IHC(P) and ELISA; may cross-react with all six known isoforms of Actin in higher vertebrates (including cytoplasmic β- and γ- Actin isoforms, skeletal, cardiac, and vascular α-Actin isoforms, and enteric γ-Actin isoform).<br>Anti-Vinculin. This antibody reacts with Human Vinculin. Based on sequence similarity, reactivity to chimpanzee, Rhesus monkey, swine, equine, mouse, rat, bovine, and chicken is expected. Immunogen sequence IRGALAEARKIAELCDDPKERDDILRSLGEI SALTSKLADLRRQGKGDSPEARALAKQV ATALQNLQTKTNRAVANSRPAKAAVHLE GKIEQAQRWIDNPTVDDRGVGQAAIRGL VAEGHRLANVMMGPYRQDLLAKCDRVD QLTAQLADLAARGEGESPQARALASQLQ DSLKDLKARMQEAM. Test material for western blotting/ immunofluorescence: HeLa cells. This Antibody was verified by Relative expression to ensure that the antibody binds to the antigen stated.<br>Goat anti-Mouse IgG, HRP-conjugated secondary - Antibody Specificity: This antibody reacts with whole molecule of mouse IgG and with the light chains of other mouse immunoglobulins. No antibody was detected against non-immunoglobulin serum proteins. The antibody has been tested by ELISA and/or solid-phase adsorbed to ensure minimal cross-reaction with human, bovine, and horse serum proteins. However, this antibody may cross-react with immunoglobulins from other species.<br>Goat anti-Rabbit IgG, HRP-conjugated secondary - Antibody Specificity: This antibody reacts with the heavy chains of rabbit IgG and with the light chains common to most rabbit immunoglobulins. No antibody was detected against non-immunoglobulin serum proteins. The product has been tested by ELISA and/or solid-phase adsorbed to ensure minimal cross-reaction with human serum proteins. However, this antibody may cross-react with immunoglobulins from other species. This antibody may cross-react with SuperBlock® Blocking Buffers.

Eukaryotic cell lines

Policy information about cell lines

Cell line source(s)	MCF10A (ATCC, ARL-10317), CAL-51 (DSMZ, ACC 302), 293FT (Invitrogen, R70007).
Authentication	DNA fingerprinting analysis was performed on CAL-51 and several isogenic clones in March 2016. MCF10A and CAL-51 parental isogenic cell lines were also validated against known karyotypes.
Mycoplasma contamination	All cell lines were routinely tested to be negative for mycoplasma.
Commonly misidentified lines (See ICLAC register)	No commonly misidentified cell lines were used.

Animals and other organisms

Policy information about studies involving animals; ARRIVE guidelines recommended for reporting animal research

Laboratory animals	Mouse Model: Athymic nude, nude (NU/J F6 HOM, #002019) The Jackson Laboratory, Female 6-8 weeks. Mice were housed with 12 h light-dark cycles at 20-26C and 30-70% humidity, in ventilated cages with five mice per cage, and constant access to food and water.
Wild animals	The study did not involve wild animals.
Field-collected samples	The study did not involve samples collected from the field.
Ethics oversight	Mice were housed and treated in accordance with NIH guidelines and protocols approved by the Institutional Animal Care and Use Committee at the Vanderbilt University Medical Center.

Note that full information on the approval of the study protocol must also be provided in the manuscript.

Flow Cytometry

Plots

Confirm that:

☒ The axis labels state the marker and fluorochrome used (e.g. CD4-FITC).

☒ The axis scales are clearly visible. Include numbers along axes only for bottom left plot of group (a 'group' is an analysis of identical markers).

☒ All plots are contour plots with outliers or pseudocolor plots.

☒ A numerical value for number of cells or percentage (with statistics) is provided.

Methodology

Sample preparation	For DNA content analysis, one million cells were prepared for flow cytometry cell cycle analysis by trypsinization and washing with PBS, followed by fixation in 70% ethanol. Cells were stained in a solution consisting of 0.1% (v/v) Triton X-100 and 20 ug/mL propidium iodide. For metabolic staining, 225 µL of a one million cell per mL cell suspension was plated into a 96-well plate, washed with FACS buffer (2% FBS in PBS), and resuspended in the appropriate stain diluted in complete medium. Cells were incubated at 37oC for 30 min, washed twice with FACS buffer before resuspension in FACS buffer.
Instrument	For DNA content analysis, samples were analyzed at the Vanderbilt Flow Cytometry Shared Resource on a 3-laser LSRSII (BD). For metabolic staining, samples were run with a Macsquant Analyzer 10 (Miltenyi Biotec).
Software	FlowJo (10.0.7) and (10.5.3).
Cell population abundance	Stop gates were set at 10,000 or 50,000 live cells for DNA content and metabolic staining analyses, respectively. Cells were not sorted.
Gating strategy	For DNA content analyses, live cells were gated, and single cells were selected by pulse processing. For metabolic staining, singlets were gated using FSC-A and FSC-H, then live cells were gated using FSC-A and SSC-A.

☒ Tick this box to confirm that a figure exemplifying the gating strategy is provided in the Supplementary Information.
